# Overview of the blood compatibility of nanomedicines: A trend analysis of in vitro and in vivo studies

**DOI:** 10.1002/wnan.1546

**Published:** 2018-12-17

**Authors:** Patricia Urbán, Neill J. Liptrott, Susanne Bremer

**Affiliations:** ^1^ Consumer Products Safety Unit, Directorate F ‐ Health, Consumers and Reference Materials, European Commission Joint Research Centre (JRC) Ispra (VA) Italy; ^2^ Department of Molecular and Clinical Pharmacology Institute of Translational Medicine, University of Liverpool Liverpool UK

**Keywords:** blood compatibility, characterization, nanomaterials, nanomedicine, preclinical, test methods

## Abstract

As nanomedicines have the potential to address many currently unmet medical needs, the early identification of regulatory requirements that could hamper a smooth translation of nanomedicines from the laboratory environment to clinical applications is of utmost importance. The blood system is especially relevant as many nanomedicinal products that are currently under development are designed for intravenous administration and cells of the blood system will be among the first biological systems exposed to the injected nanomedicine. This review collects and summarizes the current knowledge related to the blood compatibility of nanomedicines and nanomaterials with a potential use in biomedical applications. Different types of nanomedicines were analyzed for their toxicity to the blood system, and the role of their physicochemical properties was further elucidated. Trends were identified related to: (a) the nature of the most frequently occurring blood incompatibilities such as thrombogenicity and complement activation, (b) the contribution of physicochemical properties to these blood incompatibilities, and (c) the similarities between data retrieved from in vivo and in vitro studies. Finally, we provide an overview of available standards that allow evaluating the compatibility of a material with the blood system.

This article is categorized under:

Toxicology and Regulatory Issues in Nanomedicine > Toxicology of Nanomaterials
Therapeutic Approaches and Drug Discovery > Emerging Technologies
Toxicology and Regulatory Issues in Nanomedicine > Regulatory and Policy Issues in Nanomedicine

## Graphical abstract

Publications reporting blood incompatibilities for nanomedicines, *in vivo* (A) or *in vitro* (B). The most frequently reported blood adversity *in vivo* was thrombosis. Alterations on coagulation and platelets are *in vitro* endpoints corresponding to thrombosis *in vivo*; counted together, they are also the main blood incompatibility *in vitro*.

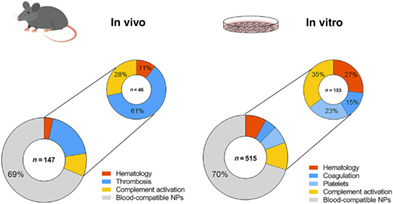

## 1 INTRODUCTION

The advantages of applying nanotechnology in the medical field have been extensively reviewed (Kaur et al., 2014) and the potential of nanomedicines raises many hopes to address unmet medical needs, as defined by the WHO in its report on priority medicines for Europe (Kaplan et al., 2013). The feasibility of translating innovative therapeutic concepts into medicinal products has been recently shown (D'Mello et al., 2017). In the United States, there is a clear increase in the number of applications for drug products seeking regulatory approval from the Food and Drug Administration's (FDA's) Centre for Drug Evaluation and Research (CDER), with more than 359 applications already submitted and each year more than 15 new applications being registered. Similarly, in Europe more than 250 clinical trials have been registered, and approximately 30 different nanotechnology‐based products have been approved (European Clinical Trials Database, 2017). Such success stories contribute to the positive market forecasts, which predict that the global nanomedicine market size could reach approximately $350.8 billion by 2025 (Grand View Research, 2017).

However, the full exploitation of nanotechnology in medicinal products still requires efforts to anticipate the upcoming challenges in translating it from the laboratory environment to clinical applications. A systematic analysis of the rate and reasons for failure of nanomedicines in preclinical and clinical development would be relevant, but is not yet available. Nevertheless, as toxicity accounts for 20% of all drug failures in clinical trials, toxic effects must be considered as potential contributors to the failure of nanomedicines (Kola & Landis, 2004). In particular, effects on the blood system are responsible for 11% of the total drug withdrawals reported by the FDA (Onakpoya, Heneghan, & Aronson, 2016). With the growing evidence of blood incompatibilities of nanomedicines in the scientific literature, we decided to perform a detailed analysis of those publications reporting blood incompatibilities of nanomedicines and nanomaterials with a potential use in biomedical applications. Different types of nanomedicines were analyzed for their toxicities to the blood system and the role of physicochemical properties was further elucidated. Especially, the particularities of the involved nanomaterial need to be understood as they might trigger unexpected adverse events. Such knowledge will support the design of nanomedicines in the research and development phase and contribute to the reduction of failure rates in clinical studies thereafter. A detailed understanding of mechanisms leading to blood toxicity could allow the selection of the most relevant and suitable test methods for screening the blood compatibility early in product development. Although a number of standard test methods have been identified, their relevance and suitability for the evaluation of the most important toxicological mechanisms leading to blood incompatibilities need to be demonstrated.

## 2 SEARCH STRATEGY

### 2.1 Blood toxicities triggered by nanomedicines

An extensive literature review was carried out using Scopus, GoPubMed and Web of Science databases to identify and collect peer‐reviewed scientific articles reporting blood toxicological effects of various types of nanomaterials with potential use in the biomedical field and nanomedicine. The working definition of nanomedicines established by the European Medicines Agency (EMA) was used in this study, described as purposely designed systems for clinical applications with at least one component at nanoscale size and resulting in definable specific properties and characteristics (Hernán Pérez de la Ossa, 2014; Quiros‐Pesudo et al., 2018). The bibliographic search covered publications until April 2017. Nanomedicines were classified in the following types: lipid‐based, polymer‐based, or inorganic nanoparticles (NPs) using the classification scheme of Wicki, Witzigmann, Balasubramanian, and Huwyler (2015).

Three blood incompatibilities were considered for in vivo studies: hematology (including hemolysis and leukocyte count), thrombosis, and complement activation; according to the recommended categories and assays proposed in ISO 10993‐4 (ISO, 2017b). For in vitro studies, the endpoints assessed were: hematology, coagulation, platelets (aggregation and/or activation), and complement activation. In addition, available information regarding the different physicochemical properties of nanomedicines, which the authors claimed to have an effect on blood toxicity was extracted, when available, from the identified publications.

For in vivo studies, the used keywords were: (nano*) AND (biomedic* or nanomed*) AND hemolysis or haemolysis AND in vivo; (nano*) AND (biomedic* or nanomed*) AND thrombosis AND in vivo; and (nano*) AND (biomedic* or nanomed*) AND complement activation AND in vivo.

For in vitro studies, the keywords used were: (nano*) AND (biomedic* or nanomed*) AND hemolysis or haemolysis AND in vitro; (nano*) AND (biomedic* or nanomed*) AND coagulation AND in vitro; (nano*) AND (biomedic* or nanomed*) AND platelet AND in vitro; and (nano*) AND (biomedic* or nanomed*) AND complement activation AND in vitro.

**Figure 1:**
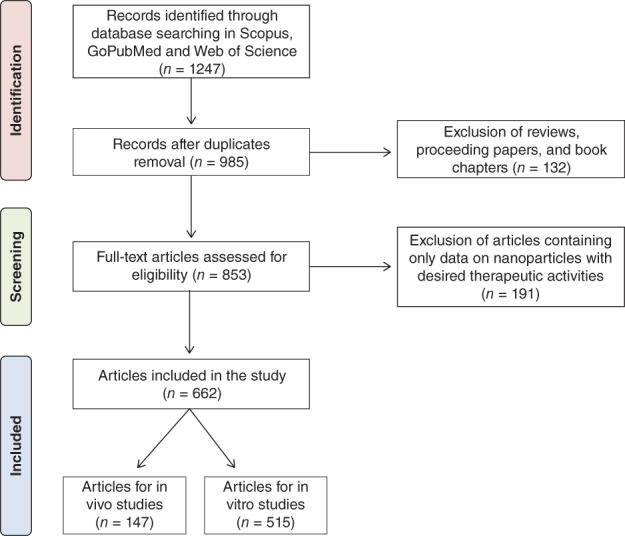
Flow diagram of the methodology used to select articles which were included in the study. It is based on the Preferred Reporting Items for Systematic Reviews and Meta‐Analyses (PRISMA). The diagram maps out the number of records (*n*) identified, included, and excluded; and the reasons for exclusion

The flow diagram of the search strategy used is shown in Figure 1. The search resulted in 1,247 records, which were collected, and identified duplicates were removed. Reviews, conference proceedings, and book chapters were excluded from the quantified results, leading to 853 screened full‐text articles. Data on nanoparticles targeting coagulation diseases or the immune system with desired therapeutic activities (e.g., procoagulant or anticoagulant effects) were excluded from our data, giving a final data set of 662 articles which were considered relevant for our study (147 articles for in vivo and 515 for in vitro studies). Information regarding the materials tested, the type of blood incompatibility and the corresponding reference to the articles used for the study are listed in Supporting Information Tables S1 and S2. Information about the blood incompatibilities reported (e.g., hematology) the effect observed (e.g., hemolysis), the type of nanoparticle causing it (e.g., inorganic nanoparticles) and where possible, the nanoparticles physicochemical properties was extracted from each article. In our analysis, a single publication with information on more than one physicochemical property would be counted more than once. The data generated in this review will be accessible from the JRC data catalogue (http://data.jrc.ec.europa.eu/).

Nanoparticles were considered hemolytic when a percentage of in vitro hemolysis above 5% was reported. Currently, there are no established pass/fail criteria for alterations in thrombosis or for a level of complement activation that is clinically relevant. Therefore, for this review, all the publications reporting an increase in the levels of complement activation or altered thrombogenicity (coagulation or platelets for in vitro studies) have been considered as positive for these endpoints. However, it should be kept in mind that a statistically significant change in clotting times does not necessarily equate to a biological relevant response, because normal hemostasis can occur even if the activity of a particular clotting factor is reduced dramatically. The present study does not take into account published literature on white blood cells; which is subject of a recently published study by the JRC (Halamoda‐Kenzaoui & Bremer‐Hoffmann, 2018a).

From the 147 screened publications for in vivo studies, 46 publications reported blood toxicities and contained information regarding the contribution of NPs physicochemical properties on the reported blood incompatibilities. For in vitro data, 153 publications reported blood toxicities and contained information regarding the contribution of each NP property on the reported toxicities. Contributions of different physicochemical properties of nanomedicines to blood incompatibilities were calculated.

We would like to note that leading to firm conclusions was challenging in this extensive review due to the heterogeneity of publicly available data on preclinical effects of nanomedicines. A lack of harmonization regarding the information available in the literature about dose‐selection, dose‐metrics, blood preparation, assay formats or species to use did not allow performing a quantitative analysis for the establishment of dose–effect relationships of physicochemical properties and adverse effects (Dobrovolskaia, Shurin, & Shvedova, 2016). Furthermore, the origin of data deriving from animal in vivo studies, human in vivo studies, and in vitro studies using blood from multiple species complicates the comparison of results obtained by different laboratories.

### 2.2 Available guidelines for blood compatibility testing

A search of available standards and guidelines for in vitro preclinical characterization of blood compatibility was performed to have an overview on available standards that can address the above‐identified endpoints. Documentary Standards from the International Organization for Standardization (ISO), ASTM International, European Committee for Standardization (CEN), International Council for Standardization in Haematology (ICSH), Clinical & Laboratory Standards Institute (CLSI), and Regulatory Agency Guidance Documents were screened. Relevant assays for regulatory purposes were identified and listed, including information regarding the type of document, the blood incompatibility addressed and the in vitro or in vivo methods described.

## 3 BLOOD TOXICITIES TRIGGERED BY NANOMEDICINES

### 3.1 In vivo blood toxicities triggered by nanomedicines

**Figure 2:**
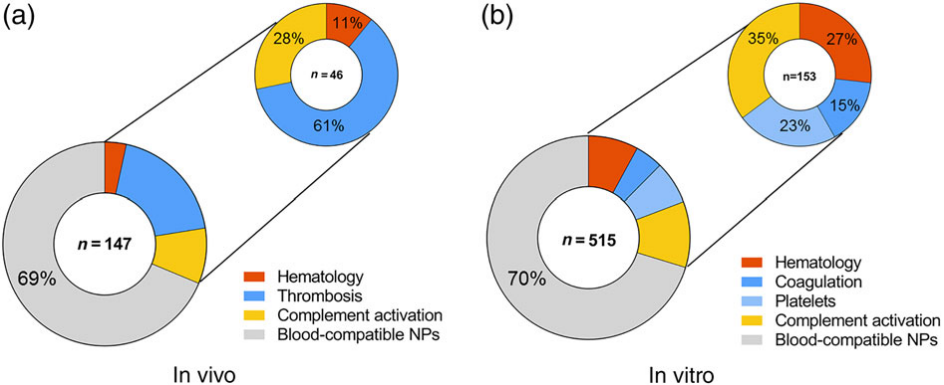
Number of publications (*n*) reporting blood incompatibilities for nanomedicines, in vivo (a) or in vitro (b). The most frequently reported blood incompatibility in vivo was thrombosis. Alterations on coagulation and platelets are in vitro endpoints corresponding to thrombosis in vivo; counted together, they are also the main blood incompatibility in vitro

In the first step of our literature review, we identified 147 articles reporting on in vivo studies that assessed adverse effects of three types of nanoparticles: lipid‐based, polymer‐based, and inorganic nanoparticles. In all, 31% of these studies (46 out of 147 publications) described blood toxicities (Figure 2a) which have been grouped according to their three main biological effects on: hematology, thrombosis, and complement activation.

Thrombosis represents the most commonly reported blood toxicity during preclinical characterization of nanomedicines in vivo, with 61% of reported cases from the total blood toxicities (28 out of 46). The remaining reports on blood toxicities were associated with undesired complement activation (28%, 13 out of 46) and alterations on hematology (11%, 5 out of 46).

Thrombogenicity is the propensity of a material to induce blood clotting and partial or complete occlusion of a blood vessel by a thrombus (a mixture of red blood cells [RBCs], aggregated platelets, fibrin, and other cellular elements). Thrombosis comprises diverse effects such as platelet aggregation, plasma coagulation, disseminated intravascular coagulation, and leukocyte procoagulant activity. Nanoparticles intended for drug delivery applications are intentionally engineered to reduce their clearance from the bloodstream and extend their systemic circulation times to increase drug delivery to a target site (Dobrovolskaia & McNeil, 2013). This extended exposure of the nanoparticles to coagulation factors and platelets may amplify adverse effects such as disseminated intravascular coagulation, which is a coagulation toxicity characterized by initial massive blood clotting, and it is increasingly being reported for cationic engineered nanomaterials (Dobrovolskaia & McNeil, 2015). Safe nanoparticles are expected to have no effect on the coagulation time. Shortening of coagulation times can induce toxicity by formation of a thrombus that could partially or completely occlude blood vessels; while an anticoagulant effect (longer coagulation times) could cause hemorrhaging (Dobrovolskaia, Aggarwal, Hall, & McNeil, 2008). Furthermore, nanoparticles may interact with and modulate the activity of various components of the coagulation system such as platelets, endothelial cells, leukocytes, and plasma coagulation factors. However, the detailed mechanisms of procoagulatory events upon blood contact with artificial surfaces are still not fully understood (Braune, Grunze, Straub, & Jung, 2013), despite this being the most reported, as well as intensely researched, blood adversity of nanomedicines in vivo.

In addition to effects leading to thrombosis, we could demonstrate that undesired complement activation was reported in 28% (13 out of 46) of the reviewed articles describing blood toxicities (Figure 2a). The complement system is a group of proteins from the innate immune system that are linked to each other in a biochemical cascade which contributes to the removal of pathogens from the body and supports cell‐mediated immunity. The activation of the complement by a pathogen or a nanostructure can be triggered by different initiation pathways involving different proteins, in which the protein C3 acts as a key player in the activation cascade (Vauthier, Persson, Lindner, & Cabane, 2011). The complement may contribute significantly to thrombosis by directly enhancing blood‐clotting properties and stimulating the inflammatory response, which in turn potentiates coagulation (Markiewski, Nilsson, Nilsson Ekdahl, Mollnes, & Lambris, 2007). Uncontrolled complement activation has been reported for several nanomedicines, including the so‐called stealth NPs such as liposomes (Wibroe & Moghimi, 2012). Low activation of complement is desired for safety reasons, since it has been proposed as being responsible for hypersensitivity reactions and the complement activation‐related pseudo‐allergy (CARPA) (Chanan‐Khan et al., 2003). CARPA is provoked by the recognition of nanomedicines by the immune system, leading to an activation of the complement cascade that could be life‐threatening. Activation of the complement is difficult to study in vivo; only an animal model based on pigs can identify immune reactive nanoparticles that may cause severe reactions in hypersensitive individuals, but its oversensitivity could lead to false positives. Furthermore, not all patients with complement activation displayed hypersensitivity in vivo. Although CARPA ceases in most patients within minutes or hours after stopping the infusion, this reaction may become life‐threatening in a minority of patients, with substantial inter‐individual variation in complement response to the same activator (Szebeni & Storm, 2015).

Only 11% (5 out of 46) of the reported blood incompatibilities in vivo were due to problems related to hematology (Figure 2a). Observed effects included alterations of complete blood count, leukocyte activation, and hemolysis (ISO, 2017b). Hemolysis is used to describe damage in the RBC membrane, leading to leakage of the iron‐containing protein hemoglobin into the blood stream. When a clinically significant drop in erythrocyte count occurs, anemia and compromised oxygen‐carrying capacity can affect the brain and other organs and be a life‐threatening condition. Intravascular hemolysis can also have effects on other blood cells compartments such as the coagulation, or the immune system (Desai, 2012; Dobrovolskaia, 2008).

### 3.2 In vitro toxicities triggered by nanomedicines

Blood toxicities evaluated by using in vitro test methods were also reviewed to correlate observed adverse effects described in vivo with available in vitro test methods (Figure 2b). The total number of reviewed publications was 515 and 30% of them described in vitro blood‐incompatible nanoparticles (153 out of 515). Interestingly, the total percentage of reported incompatibilities was almost the same, around 30%, for in vivo and in vitro assays. Four endpoints were considered relevant when testing blood toxicities in vitro: hematology (including hemolysis and leukocyte count), coagulation, platelets (aggregation and/or activation), and complement activation.

Complement activation was the most commonly found blood adversity, accounting for 35% (54 out of 153) of the reported incompatibilities (Figure 2b). Several groups have reported a good correlation between the observed complement activation both in vitro and in vivo for various engineered nanomaterials (Chanan‐Khan et al., 2003; Dobrovolskaia & McNeil, 2013).

In all, 27% of the reported incompatibilities corresponded to alterations in hematology (41 out of 153, Figure 2b). Results from the in vitro hemolytic activity are likely an early indicator of the interaction between the nanoparticle and the cell membrane and toxic behaviour towards cells. The hemolytic potential of nanoparticles is a crucial initial test for assessing biocompatibility in vitro, and hemolysis has been reported to have a good correlation in vitro–in vivo (Dobrovolskaia & McNeil, 2013).

Finally, 23% of the reported incompatibilities corresponded to changes in platelet function (35 out of 153) and 15% to an unbalanced coagulation process (23 out of 153) (Figure 2b). Alterations in the platelets and coagulation cascade are the in vitro endpoints corresponding to thrombosis in vivo. Platelet aggregation in vitro is often used as a marker of the thrombogenic properties of the test material. Platelet aggregation has been described as requiring the activation of glycoprotein integrin receptor GPIIb/IIIa, and different sizes of nanoparticles might activate this receptor through different pathways (Radomski et al., 2005). The imbalance in the coagulation process could be explained by the fact that proteins from the coagulation cascade are often found in the corona around nanoparticles (Sanfins, Augustsson, Dahlbäck, Linse, & Cedervall, 2014), and this nanoparticle–protein interaction can induce conformational and functional alterations in the proteins that could be responsible for the observed effects on the blood coagulation cascade. Currently, only a combination of in vitro assays targeting platelets and coagulation pathways seems promising to predict the procoagulant and anticoagulant properties of a nanoparticle.

## 4 GENERAL BLOOD TOXICITIES ASSOCIATED WITH EACH TYPE OF NANOPARTICLES

**Figure 3:**
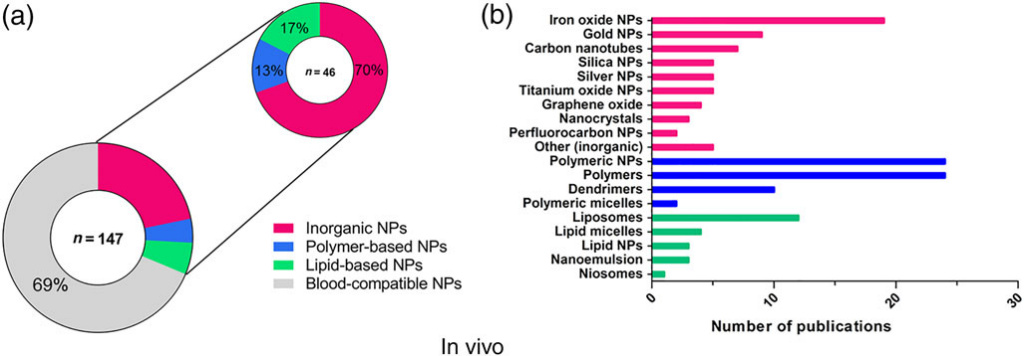
General blood incompatibilities linked to the types of nanomedicines in vivo. In all, 46 articles out of the 147 screened publications reported blood incompatibilities, and inorganic nanoparticles were most frequently associated with blood toxicities (a). Types of nanomedicines and nanomaterials with potential use in biomedicine retrieved from the screened publications (color—coded) and number of publications found for each type of nanomaterial. For inorganic NPs only one publication was found for some NPs, which were grouped under the category *Other (inorganic)* (b)

To further understand the reported trends for blood toxicities related to nanomedicines, the type of nanoparticle causing blood incompatibilities was identified in the reported cases. For in vivo effects, the majority of blood toxicities were linked to inorganic nanoparticles, with 70% (32 out of 46) of the reported adversities (Figure 3a). Lipid‐based nanoparticles triggered 17% (8 out of 46) of the reported cases. The best compatibility was achieved by polymer‐based nanoparticles, with only 13% (6 out of 46) of the reported cases. The different types of nanomedicines and nanomaterials with potential use in the biomedical field, which were included in each category of nanomedicines for in vivo studies, are described in Figure 2b. The number of articles per nanomaterial is also presented in the graph. The highest number of articles was found for polymeric NPs, followed by polymers with applications in the biomedical field, iron oxide NPs, liposomes, dendrimers, gold NPs, and carbon nanotubes. The category “Other (inorganic)” included inorganic nanoparticles such as carbon nanocapsules, hydroxyapatite NPs, nanodiamonds, polystyrene NPs, and quantum dots, and was represented by only one publication.

**Figure 4:**
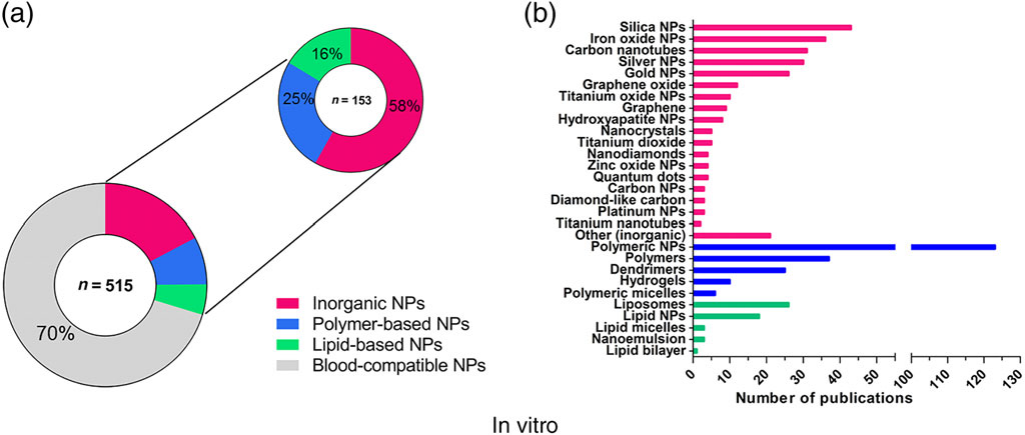
General blood incompatibilities linked to the different types of nanomedicines in vitro. Inorganic NPs were the type of nanomedicines most frequently associated with blood incompatibilities (a). The different types of nanomaterials included in each type (color—coded), and the number of publications found for each nanomaterial in vitro is shown. The highest number of publications were found for polymeric NPs (*n* = 123) (b)

In agreement with the data extracted from in vivo experiments, blood incompatibilities were most frequently reported for inorganic nanoparticles using in vitro tests, representing 58% (89 out of 153) of blood toxicities found in the literature (Figure 4a). Polymer‐based nanoparticles accounted for 25% (39 out of 153) of the total blood toxicities and lipid‐based NPs for 16% (25 out of 153). The number of publications found for each type of nanomaterial included in the three categories is also presented (Figure 4b). The highest number of publications was found for polymeric NPs, followed by silica NPs, polymers, iron oxide NPs, carbon nanotubes, silver NPs, gold NPs, liposomes, dendrimers, and lipid NPs. In order to simplify the data visualization, inorganic NPs which corresponded to only one publication were grouped in the category “Other (inorganic)”, and included: aluminum oxide NPs, apatite NPs, carbon dots, cobalt ferrite NPs, graphene oxide NPs, halloysite nanotubes, hydroxide NPs, or silicon nanowires.

## 5 ASSOCIATION BETWEEN SPECIFIC BLOOD TOXICITIES AND TYPE OF NPS

As a next step, the particular blood toxicities that were reported for each type of nanomaterial were analyzed in more detail to see if specific toxicities could be related to certain types of nanoparticles.

### 5.1 In vivo toxicities

**Figure 5:**
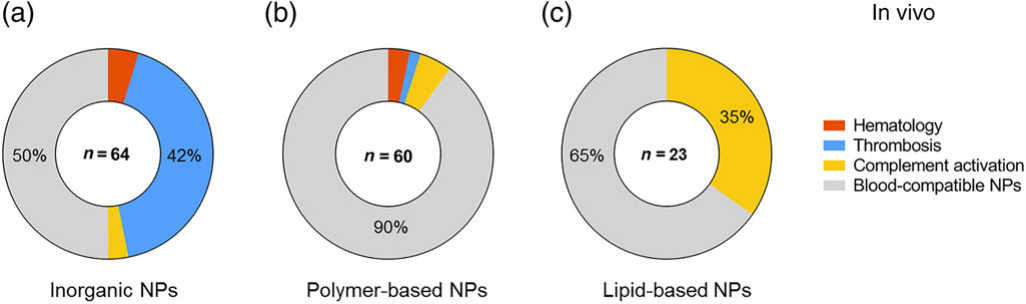
Number of publications reporting blood adverse effects in vivo for inorganic (a), polymer‐based (b) and lipid‐based NPs (c). The main adversity associated with inorganic NPs in vivo was thrombosis. Polymers did not show relevant blood toxicities, and lipid‐based NPs were linked with undesired complement activation

In 50% of the articles describing inorganic NPs (32 out of 64), the nanoparticles were claimed to be blood‐compatible in in vivo studies (Figure 5a). The main adversity associated with these nanoparticles was thrombosis, which had been reported in 42% of the articles (27 out of 64). Only a few cases of hematology‐related toxicity were associated with inorganic nanoparticles (5%, 3 out of 64), and complement activation (5%, 3 out of 64).

The majority of the articles reporting on polymer‐based NPs showed excellent blood compatibility (90%, 54 out of 60). However, some publications demonstrated toxic effects on the blood system: 3% for hematology (2 out of 60), 2% for thrombosis (1 out of 60), and 5% for complement activation (3 out of 60) (Figure 5b).

For lipid‐based nanomedicines, 67% of the publications (16 out of 24) reported that these NPs were blood‐compatible (Figure 5c). The dominant reported effect in vivo for this type of nanoparticles was complement activation, with 33% reporting undesirable activation (8 out of 24). Lipid‐based nanoparticles resemble pathogenic microbes and subcellular organelles in size and shape, which might be the biological rationale behind the complement activation (Szebeni, Muggia, Gabizon, & Barenholz, 2011). No cases were found reporting hematology‐associated toxicity or thrombosis for lipid‐based NPs.

### 5.2 In vitro toxicities

**Figure 6:**
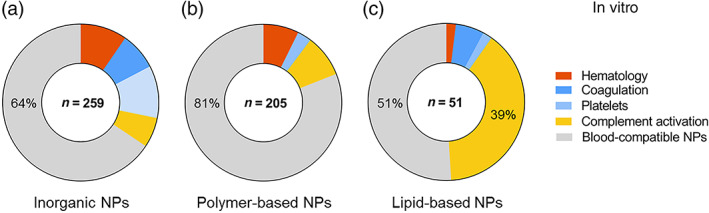
Number of publications reporting blood toxicities in vitro for inorganic (a), polymer‐based (b) and lipid‐based NPs (c). Alterations on coagulation and platelets counted together were the main adversity for inorganic NPs in vitro. Polymer‐based NPs were the most compatible NPs. Complement activation was the most frequently reported blood toxicity associated with lipid‐based NPs

Of 259 articles found for inorganic NPs, 170 concluded that the assessed NPs were blood‐compatible in vitro (64%) (Figure 6a). Hemostasis‐related alterations were the main effect observed also for inorganic NPs in vitro, which included imbalances in the coagulation cascade (20 out of 259, 8%) and modifications on the functionality of platelets (platelets aggregation and/or activation in 28 out of 259, 11%). Both effects can be associated with thrombosis in vivo. Toxic effects on hematology and complement activation were also reported for inorganic nanoparticles. In all, 10% of the publications (25 out of 259) identified effects on hemolysis and leukocyte count for this category of nanoparticles and 6% of the publications (16 out of 259) described the activation of the complement system.

The majority of polymer‐based NPs were compatible with the blood system according to the performed in vitro studies (81%, 166 out of 205). The main observed adversity was complement activation (Figure 6b) described in 9% of the reported studies (18 out of 205). Other blood toxicities associated to polymer‐based NPs were effects on hematology (15 out of 205, 7%) and alterations on platelets function (6 out of 205, 3%). We did not find any publication reporting an effect of these NPs on the coagulation cascade.

In all, 51% of the articles (26 out of 51) reported that lipid‐based NPs were blood‐compatible (Figure 6c). Complement activation was the main adversity associated with type of nanoparticles with 39% of reported studies (20 out of 51), which was in agreement with the data obtained from the in vivo studies. It has been increasingly recognized during the last decade that a major cause of acute infusion reactions to liposome‐ and micelle‐based nanomedicines is their ability to activate the complement system. Alterations on the hemostasis were also reported for lipid‐based nanoparticles in vitro, even if no effects in thrombosis in vivo had been found (3 out of 51, 6% for coagulation and 1 out of 51, 2% for altered platelet function). Effects on hematology were only described in 1 publication out of 51 (2%).

Our results indicate that some nanomaterials have more frequently been linked with specific blood incompatibilities in literature: the main toxicity associated with inorganic nanoparticles is thrombosis, whereas complement activation is the most reported blood toxicity linked to lipid‐based nanoparticles. Only a few cases of blood toxicities were linked to polymer‐based nanoparticles. This knowledge could be used to raise the regulatory awareness of specific adverse reactions that need to be addressed, because some categories of nanomedicines may be more interactive with a particular biological system. Furthermore, this information can also support nanomedicines developers in their design of safe and efficacious nanomedicinal products with improved blood compatibility.

## 6 ASSOCIATION BETWEEN PHYSICOCHEMICAL PROPERTIES AND TOXICITIES

A link between the physicochemical properties of the nanomaterial and the toxicological outcome at the cellular level has already been proposed in literature (Hall, Dobrovolskaia, Patri, & McNeil, 2007; Mayer et al., 2009); but only a limited amount of preclinical in vivo data with effects on the blood system has been published. Different characteristics such as size, particle structure, shape, and surface chemistry have been suggested as drivers of the biological behaviour of nanoparticles (Nel, 2013; Fadeel, 2013; Fadeel & Garcia‐Bennett, 2010). Additionally, the analysis of the protein corona is gaining importance to generate a holistic view of the physicochemical characteristics of nanoparticles that may influence their compatibility. For instance, FDA's CDER has recently published a draft guidance on drug products that contain nanomaterials (as active ingredients, carriers, or inactive ingredients) (US FDA Center for Drug Evaluation and Research (CDER); Center for Biologics Evaluation and Research (CBER), 2017). This guidance acknowledges the fact that after entry into the systemic circulation, nanomaterials can affect the distribution, exposure–response profile, and the residence time of an active ingredient. These changes may be partly due to the interaction of nanomaterials with plasma proteins (and formation of a protein corona), which may endow nanomaterials with new biological properties. Therefore, the guidance for drug products containing nanomaterials recommends testing of plasma protein binding.

**Figure 7:**
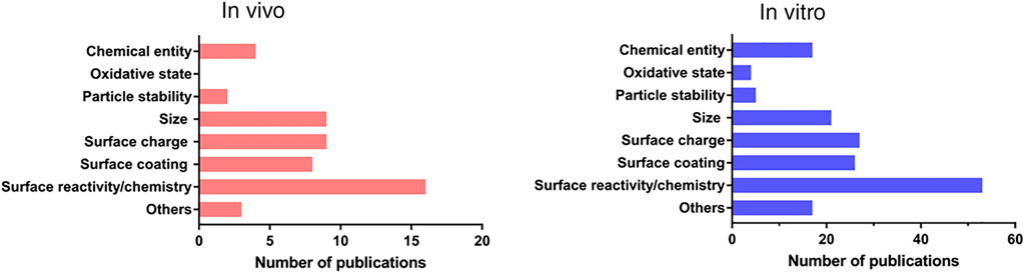
Number of identified publications associating key physicochemical properties of nanomedicines with general blood toxicities in vivo (left) and in vitro (right). Surface‐related properties were the most reported properties having an influence on blood adversities. The category *Others* includes properties such as shape, type of drug encapsulated, porosity, roughness, or elasticity

In order to gain further understanding of blood compatibility of nanomedicines, we tried to identify trends in the physicochemical properties that have an impact on blood toxicity. Information regarding the contribution of specific physicochemical properties of NPs on the reported adversities in vitro and in vivo was retrieved from the screened publications. The most relevant physicochemical properties described in the reviewed articles that have an impact on general blood adversities are shown in Figure 7. Surface‐related properties (such as surface chemistry, surface coating, and surface charge) are most commonly reported to have a key role on blood toxicities. These results indicate that these properties are important in the interaction of nanomedicines with blood cells and can therefore be seen as initiators of the biological effect. Size and chemical entity have also been identified as important contributors for undesired effects on blood cells, and other properties such as shape, curvature, or type of encapsulated drug can also add to the observed blood toxicities.

**Figure 8:**
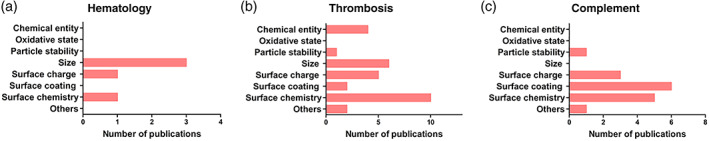
Number of publications associating specific physicochemical properties of nanomedicines with each of the studied endpoints for blood toxicity in vivo: Hematology (a), thrombosis (b), and complement activation (c)

Understanding the influence of specific properties of nanomedicines on each compartment of the blood system is crucial for developing nanomaterials used in biomedical applications. For this purpose, the contribution of the reported physicochemical properties to each of the studied endpoints was assessed individually. Results obtained for in vivo data are shown in Figure 8; it has to be noted that there was a limited number of available publications related to in vivo blood compatibility of nanomedicines. Size has been described as the main contributor to adverse effects on hematology in vivo, interestingly; size was not described in literature as having a role for toxicity on the complement system. The type of chemical entity of the nanomedicine and surface chemistry were shown to have the largest influence on thrombosis, followed by size and surface charge. Surface‐related properties such as surface chemistry, surface coating, and surface charge were the most frequently reported properties to contribute to complement activation in vivo.

**Figure 9:**
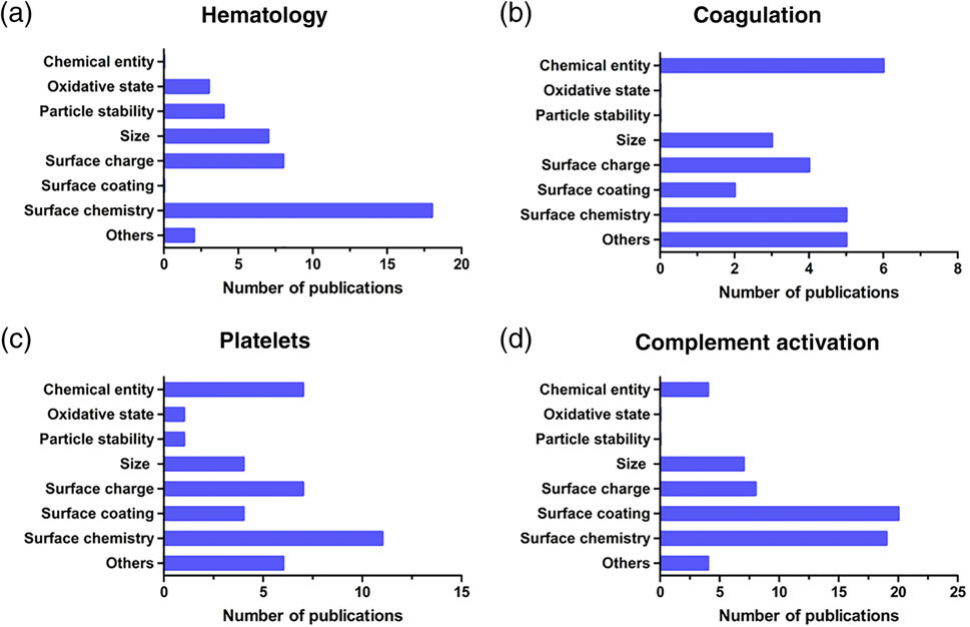
Number of publications associating specific physicochemical properties of nanomedicines with each of the studied endpoints for blood toxicity in vitro: Hematology (a), coagulation (b), platelets (c), and complement activation (d)

The contribution of the reported physicochemical properties to each of the in vitro endpoints individually is shown in Figure 9. Surface characteristics such as surface chemistry and charge were identified as key properties causing altered effects on hematology (Figure 9a). Surface chemistry and, therefore, surface reactivity determine the interaction of nanomedicines with RBCs. For instance, the interaction of dendrimers with the RBC membrane due to electrostatic attraction was shown to lead to different outcomes depending on surface chemistry of the nanoparticles, with higher hemolysis reported for more positive dendrimers (Domański, Klajnert, & Bryszewska, 2004; Malik et al., 2000). Other studies reported that blocking the surface reactive groups of the particles reduces their interaction with the membranes of RBCs and therefore their potential hemolytic activity (Zhao et al., 2011). Size, particle stability, and oxidative state were also identified as relevant contributors to blood toxicity. Size was the main contributor to hematology toxicities in vivo; however, the exact mechanism for erythrocyte membrane damage is not clear and some publications describe contradictory results. Some of the claimed modes of action are: the formation of pores in the RBC membrane by nanoparticles leading to osmotic lysis, strong local deformation causing the destruction of RBCs or the oxidative deterioration of membranes by reactive oxygen species or lipid peroxidation (Huang et al., 2016). Other properties; more specifically, the shape of the nanomedicine, have also been suggested as contributors to this toxicity (Li et al., 2008).

Interactions of nanomedicines with coagulation factors, platelets, and endothelial cells may all contribute to undesirable coagulation‐mediated toxicities. Protein adsorption onto the nanoparticle surface is one of the first events occurring when nanoparticles reach the blood stream. It has been found that plasma proteins such as albumin, apolipoprotein, complement, or fibrinogen can adsorb on the surface of nanoparticles and exhibit structural changes (Deng, Liang, Toth, Monteiro, & Minchin, 2013; Lee, Choi, Webster, Kim, & Khang, 2014). The adsorbed proteins depend on nanoparticle physicochemical properties such as size, shape, curvature, hydrophobicity, and surface charge, (Lundqvist et al., 2017) and on the composition of the biological media. The overall protein corona composition fluctuates with time due to proteins having different affinities to nanoparticles (Setyawati, Tay, Docter, Stauber, & Leong, 2015). The type and amount of proteins adsorbed on the nanoparticles influence the interactions of engineered nanomaterials with cells and their subsequent outcomes, playing an important role on nanoparticle biocompatibility (Aggarwal, Hall, McLeland, Dobrovolskaia, & McNeil, 2009; Fadeel, 2013). The adsorption of certain plasma proteins may induce adverse effects on the blood system such as imbalance of the coagulation cascade or complement activation (Dobrovolskaia, 2009). However, the mere presence of a specific protein on the particle surface does not imply that the biological function of that protein will be activated or changed (Dobrovolskaia et al., 2014; Mayer et al., 2009). For instance, complement and fibrinogen adsorption on nanoparticles does not lead to a level of depletion of these proteins, as they are highly abundant in plasma (Deng, Liang, Monteiro, Toth, & Minchin, 2011; Deng et al., 2013).

Our review confirmed previous findings that had identified physicochemical properties such as size, surface charge, and surface chemistry as relevant for the observed alterations on the coagulation cascade (Ilinskaya & Dobrovolskaia, 2013) (Figure 9b). Surface chemistry includes aspects such as the density of surface groups or the presence of targeting moieties (Ilinskaya & Dobrovolskaia, 2013) which might also play a role in coagulation‐mediated blood toxicity (Guildford et al., 2009). Interestingly, the type of chemical entity was the most reported property for coagulation. This result is explained in the literature based on the uniqueness of each nanoparticle, as the interaction with various components of the coagulation system may even differ for nanomaterials of the same category, but, for example, of different size. In general, charged and hydrophobic materials are expected to be more reactive, and often the activation of coagulation occurs through noncanonical pathways when these properties are involved. The literature reviewed here indicated that procoagulant processes can also be induced by other intrinsic physicochemical properties of the materials such as porosity, roughness, charge density, elasticity, or surface energy (Thevenot, Hu, & Tang, 2008).

Platelets are very sensitive to changes in the blood microenvironment, and different alterations on the platelets function have been reported such as aggregation*,* degranulation, changes in shape, and the expression of activation markers (Dobrovolskaia et al., 2013; Radomski et al., 2005). As already seen in in vivo studies, in vitro studies also indicated that surface‐related properties are the main contributor to platelets alterations (Figure 9c). In addition, chemical entity and the size of the nanoparticles have been described as important parameters (Guidetti et al., 2012; Guildford et al., 2009; Laloy et al., 2014; Shrivastava et al., 2009). Other properties such as surface roughness and the conformation of the polymer on the surface of the nanoparticle might play a role on the development of the reported toxicities.

In addition, surface‐related properties can also trigger an undesired activation of the complement system. Parameters such as surface charge, chemistry and/or coating play also a role in complement activation (Figure 9d) (Dobrovolskaia, 2008; Reddy et al., 2007; Vonarbourg et al., 2006). Surface‐camouflaging elements such as polyethylene glycol, poloxamer, and poloxamine coatings may avoid recognition of nanoparticles by the immune cells (Csaba, Sánchez, & Alonso, 2006). But coating does not completely prevent complement activation. For example, it has been reported that human complement activation is independent of dextran‐coating modifications for inorganic nanoparticles (Wang et al., 2016). Our literature review indicates that size and type of chemical entity must also be considered as key contributors to complement activation. Liposomes and certain carbon nanotubes are, due to their size, within the spotlight of immune surveillance, while other types of nanoparticles fall outside immune recognition (Szebeni, 2012). Other properties of liposomes such as vesicular lipid composition, bilayer packaging, surface characteristics, and morphology can contribute to complement activation (Moghimi & Hamad, 2010). Activation of the complement can be desirable in the case of nanoparticle‐carried vaccines, where it has been shown that the generation of the antigen‐specific immune response can be dependent on complement activation by nanoparticle (Reddy et al., 2007). Therefore, the tendency of a nanoparticle to activate the complement system may be beneficial or adverse depending on the intended application of the nanomedicine. Currently, multiple physicochemical parameters are discussed as initiating events for complement activation, hence, future standardized in vitro tests assessing the complement activation can assist product development.

The observed trends for the role of different physicochemical properties in in vivo and in vitro toxicity showed similarities for some of the studied endpoints. For instance, thrombosis in vivo has been associated with NPs properties such as surface chemistry, size, surface charge, and chemical entity. These properties were also identified as key physicochemical parameters from in vitro data related to alterations in the coagulation and platelets. Regarding complement activation, surface‐related properties have been identified as critical, in both in vitro and in vivo studies. But there are divergences in the information retrieved from in vivo and in vitro studies for the hematology endpoint. In vitro data identifies surface chemistry as the main contributor to alterations in hematology, whereas, in vivo data points to size as a critical parameter. However, the low number of publications available for in vivo studies, especially for studies of the hematology endpoints, means that additional studies would be relevant to confirm this conclusion for in vivo.

As already described in literature, preclinical testing has to identify potential safety concerns, but the extrapolation of findings from in vivo toxicity assays to humans is often challenging (Dobrovolskaia & McNeil, 2013). Humanized in vitro test methods assessing, for example, blood compatibility could be valuable tools to identify potential hazards early during product development. The number of publications reporting on in vitro data as well as their similarities with in vivo data indicates the usefulness of in vitro testing in biomedical research (Figure 7). However, the relevance and reliability of such in vitro test methods for regulatory decision‐making has to be demonstrated.

## 7 AVAILABLE GUIDELINES FOR BLOOD COMPATIBILITY TESTING

Evaluation of the quality, safety, and efficacy of nanomedicines is based on the existing regulatory framework for medicinal products. But since available guidelines were mainly developed for small molecules, their applicability to nanomaterials needs to be evaluated (Halamoda‐Kenzaoui, Holzwarth, Roebben, Bogni, & Bremer‐Hoffmann, 2018b). The Committee for Medicinal Products for Human Use of the European Medicines Agency has released some reflection papers to summarize the current status of regulatory considerations when assessing a product that is nano‐enabled (EMA/CHMP, 2013a, 2013b, 2013c).

A list of available guidelines and standards containing relevant information to evaluate blood compatibility of pharmaceuticals and medical devices during the preclinical phase is shown in Table 1. Initial information on side effects of nanomedicines on the hematological system can be retrieved during in vivo standard toxicity studies (EMA/CHMP, 2010; ICH, 2009). Methods proposed in these guidelines include: complete cell count, the evaluation of erythrocytes morphology, determination of hematocrit and hemoglobin concentration, and gross examination. When additional testing is required with respect to clinical pathology, the CPMP/SWP/1042/99 Rev 1 Corr guideline (EMA/CHMP, 2010) states that the clinical pathology and the parameters measured will be specific to the species used, referring also to the literature for recommendations regarding core tests and standard sampling intervals (Weingand et al., 1996).

**Table 1:** Available standards and guidelines for the assessment of blood compatibility. Information about the endpoint covered by each document and the methods included is shown

Endpoint	Document code	Title of the document	Method	In vivo*/*in vitro
Hematology	CPMP/SWP/1042/99 Rev 1 Corr	Guideline on repeated dose toxicity	Complete blood count Evaluation of RBC morphology Hemoglobin concentration Hematocrit	In vivo
	ICH M3(R2)	Guidance on nonclinical safety studies for the conduct of human clinical trials and marketing authorization for pharmaceuticals	Complete blood count	In vivo
	ICH‐S8	Immunotoxicity studies for human pharmaceuticals S8	Hematological changes (total and absolute differential leukocytes counts)	In vivo
	ISO 10993‐4	Biological evaluation of medical devices—Part 4: Selection of tests for interaction with blood	Complete blood count	In vivo
			Hemolysis Leukocyte activation (ELISA)	In vitro
	ASTM E2524‐08	Standard test method for analysis of hemolytic properties of nanoparticles	Hemolysis	In vitro
	ASTM F2888‐13	Standard test method for platelet leukocyte count	Leukocyte count	In vitro
Thrombosis	CPMP/SWP/1042/99 Rev 1 Corr*	Guideline on repeated dose toxicity	Gross examination	In vivo
	ICH M3(R2)	Guidance on nonclinical safety studies for the conduct of human clinical trials and marketing authorization for pharmaceuticals	Gross examination	In vivo
	ICH‐S8	Immunotoxicity studies for human pharmaceuticals S8	Gross examination	In vivo
	ISO 10993‐4	Biological evaluation of medical devices—Part 4: Selection of tests for interaction with blood	Gross examination (lung, kidney) Flow reduction Light microscopy/scanning electron microscope (SEM)	In vivo
			Measure of blood plasma levels of coagulation indicators by ELISA Partial thromboplastin time Platelet counting Platelet activation markers measurement	In vitro
	ASTM F2382—17e1	Standard method for assessment of intravascular medical device materials on partial thromboplastin time (PTT)	Partial thromboplastin time	In vitro
	ASTM F2888‐13	Standard test method for platelet leukocyte count	Platelets count	In vitro
Complement activation	ISO 10993‐4	Biological evaluation of medical devices—Part 4: Selection of tests for interaction with blood	Assessment of complement factors plasma levels (C3a, SC5b‐9) by ELISA	In vitro
	ASTM F 1984—99	Standard practice for testing for whole complement activation in serum by solid materials	Total hemolytic activity of the classical pathway (CH 50)	In vitro

The International Council for Harmonisation of Technical Requirements for Pharmaceuticals for Human Use (ICH) has released the ICH S8 guideline, which contains recommendations for the evaluation of the potential immunotoxicity of human pharmaceuticals (ICH, 2005). These recommendations for nonclinical testing apply to new pharmaceuticals intended for use in humans, and therefore, to new nanomedicines. The investigation of hematological changes (e.g., leukocytopenia or granulocytopenia) and gross examination to indicate the presence of thrombus is recommended in the ICH S8 guideline. Complement assays, which are relevant for the detection of hypersensitivity or infusion reactions are not yet included, although such adversities have already been linked with nanomedicines (Chanan‐Khan et al., 2003; Giannakou et al., 2016; Szebeni, Muggia, Gabizon, & Barenholz, 2011; Szebeni, 2012).

The blood compatibility of biomaterials contained in medical devices is evaluated in conformity with the ISO 10993‐4 “Selection of tests for interaction with blood” (ISO, 2017b), which was published in 2002 and the most recent update is from 2017. This standard is the most complete available source of information for evaluating the interactions of a material with blood, and contains a list of suggested tests. These test methods are divided as: effect of materials on blood cells (Hematology), platelets and coagulation factors (thrombosis), and complement system. There are numerous methodologies for the testing of the impact of nanoparticles on blood coagulation cascades. The use of some assays commonly used in literature, such as measuring activated partial thromboplastin time (aPTT), prothrombin time, and thrombin time are not recommended by ISO 10993‐4; since they do have some limitations, for example, they assess coagulation in isolation, whereas in vivo these pathways are closely interlinked (Curry & Pierce, 2007). Additionally, these assays include an activator (e.g., aPTT assay), which could mask the potential activation caused by the nanoparticles themselves. Comparative assays such as the PTT assay, which does not include an activator, are recommended in the ISO 10993‐4 guidelines. However, additional supplementary tests might be necessary to further minimize the hazard of blood toxicities (Braune et al., 2013; Van Oeveren, 2013). A limitation of the ISO 10993‐4 is its lack of acceptance criteria and a classification to relate obtained blood toxicity results to clinical disorders. Currently, the only criteria available is the percentage hemolysis to classify a biomaterial in nonhemolytic (0–2%), slightly hemolytic (2–5%), or hemolytic (>5%).

The recently published ISO 10993‐22: Guidance On Nanomaterials (ISO, 2017a) describes general considerations for the biological evaluation of medical devices that are composed of, contain or generate nanomaterials. This standard details several pitfalls identified when testing nanomaterials and highlights the importance of proper physicochemical characterization to understand their behaviour in biological systems. Some of the key properties proposed are: chemical entity, purity, objects size and size distribution, aggregation and agglomeration state, shape, surface area, surface chemistry, or surface charge. Some examples of measurement methods for the proposed key physicochemical characteristics are listed. However, the standard acknowledges that appropriate tools and methods for the evaluation of nanomaterials are still under development and it does not include specific methods for blood compatibility assessment of nano‐enabled products. It is still under debate which supplementary assays need to be performed to enhance our understanding of blood compatibility of new materials for blood‐contacting applications (Braune et al., 2013).

As shown in Table 1, other standards that should be considered for blood compatibility are ASTM standards assessing the hemolysis, leukocyte count, coagulation, and complement activation (ASTM E2524‐08, 2013; ASTM F2382‐04, 2004; ASTM F1984‐99, 2013; ASTM F2888‐13, 2013). ASTM E2524‐08 (2013) is the only standardized test method which has been adapted to nanomaterials and it focusses on the assessment of the hemolytic properties of nanomaterials. The International Council for Standardization in Haematology (ICSH) has published several guidelines and recommendations for hematology (Briggs et al., 2014; Kratz et al., 2017; Roussel, Davis, Fest, & Wood, 2012) and platelet counting (Klee et al., 2001). The Clinical and Laboratory Standards Institute (CLSI) has also made an effort to implement clinical laboratory testing standards in the area of blood compatibility. CLSI standards are mainly focused in the assessment of alterations in the coagulation cascade (CLSI, 1997, 2001, 2008a) and platelet function (CLSI, 2008b). However, most of the currently available in vitro tests have been developed or validated for small molecules.

Most of these assays have not been challenged for their use with nanomaterials, and their suitability for nanoparticles has still to be proven. The evaluation of current standards for their suitability to assess also nanomaterials is highly relevant because potential interference of nanoparticles with test reagents have to be considered. For instance, some inorganic nanoparticles have high absorbance at different visible wavelengths, interfering with the readout (and result) of the performed assays (Laloy et al., 2012). It has already been reported that nanoparticle may interfere with the hemolysis assay proposed in ASTM E2524‐08 standard through several mechanisms (Choi, Reipa, Hitchins, Goering, & Malinauskas, 2011; Dobrovolskaia, 2008). ISO 10993‐22 recommends specific attention to the reproducibility, reliability, and sensitivity of the methods before arriving at any conclusions regarding the blood compatibility of nano‐objects, due to the potential in vitro tests interferences.

A key for a smooth translation of nanomedicines into the clinical applications is a good understanding of information requirements related to the impact of the nano‐specific properties on the quality and safety of the product. A robust, standardized in vitro blood compatibility testing battery can enable the detection, and subsequent elimination of undesired and excessive material‐induced blood adverse events at an early stage of nanomedicines development. Special emphasis should be given to the identification of in vitro tests that have the potential to identify toxic effects triggered by the nano‐specific properties of the formulation. Whilst these assays may not have been established only for nanomedicines, it is important for developers to have a resource of assays that have been demonstrated to work for nanomaterials. The strength of the nanomedicine field is the heterogeneity of the materials that are being produced for this purpose; however, this makes their assessment particularly challenging. A panel of standardized assays will support developers in assessing their materials and allow them to determine the most suitable testing of their materials. Furthermore, predictive in vitro methods can optimize the development of the candidate drugs by reducing the experimental time and costs in preclinical development. The preconditions for the translation of nanomedicines into clinical applications are promising, as the regulatory community is well aware of upcoming challenges and several international projects such as the European Nanomedicine Characterisation Laboratory (http://www.euncl.eu/), US Nanomedicine Characterization Laboratory (https://ncl.cancer.gov/), *REFINE* (http://refine-nanomed.com/), PATROLS (https://www.patrols-h2020.eu/), and BIORIMA (https://www.biorima.eu/) are currently addressing the need to develop standardized test methods for the most relevant information requirements available for nanomaterials safety assessment.

## 8 CONCLUSION

This review presents the analysis of an extensive literature review which was carried out using Scopus, GoPubMed and Web of Science databases to identify peer‐reviewed scientific articles reporting blood toxicological effects of various groups of nanomaterials. In vivo and in vitro studies were taken into account in our study. In total, 146 papers of interest analyzing hematology, thrombosis, and complement activation in vivo were considered. In addition, 515 publications evaluating hematology, coagulation, platelets, and complement activation in vitro have been identified as relevant for our investigation.

We were able to demonstrate trends related to the nature of the most frequently occurring blood incompatibilities such as thrombogenicity and complement activation. Thrombogenicity was mainly associated with inorganic NPs (reported in 42% of the screened publications in vivo) and complement activation was associated with lipid‐based NPs (in 33% of the publications for this type of NPs in vivo). This trend was also confirmed for data obtained from in vitro studies. Interestingly, only a few cases of blood toxicities were linked to polymer‐based nanoparticles. We also investigated the contribution of physicochemical properties to blood incompatibilities. Surface‐related properties (such as surface chemistry, surface coating, and surface charge) were the most frequently reported to have a key role on general blood toxicities. Furthermore, we also identified the role of particular physicochemical properties on each of the assessed endpoints since some types of nanomedicines may be more interactive with a particular biological system.

We believe that this knowledge is important to raise regulatory awareness on potential blood incompatibilities that are relevant for the evaluation of the next generation of nanomedicines. The information gained from this study will help to prioritize methods for further standardization. The association of physicochemical properties with biological effects elucidated the need for standardized methods analyzing specific nanomaterial‐related properties. In particular, surface‐related properties were related to various blood incompatibilities such as thrombosis or complement activation. In order to further analyze which standards are needed, an overview of already existing standards for the evaluation of blood compatibility has been included in this study. However, their adequacy to analyze nanomaterials has not been proved yet.

Finally, the knowledge on particular blood incompatibilities depending on the type of nanomaterial as well as the availability of suitable methods will support the establishment of tailor‐made test strategies for designing efficacious and safe nanomedicines with improved blood compatibility.

## CONFLICT OF INTEREST

The authors have declared no conflicts of interest for this article.

## DISCLOSURE OF INTERESTS

Any opinions expressed in this publication are those of the authors only, and this paper does not represent an official position of the European Commission.

## RELATED WIREs ARTICLES

The effects of nanomaterials on blood coagulation in hemostasis and thrombosis

Nanomedical engineering: shaping future nanomedicines

Nanomaterial standards for efficacy and toxicity assessment

## Supporting information


**Table S1**. List of publications used for in vivo analysis. Information regarding the test category, the category of material, the type of material and the reference to the article is shown.

**Table S2**. List of publications used for in vitro analysis. Information regarding the test category, the category of material, the type of material and the reference to the article is shown.

## References

[wnan1546-bib-0013] Chapman, S. , Dobrovolskaia, M. , Farahani, K. , Goodwin, A. , Joshi, A. , Lee, H. , … Yang, L. (2013). Nanoparticles for cancer imaging: The good, the bad, and the promise. Nano Today, 8(5), 454–460. 10.1016/j.nantod.2013.06.001 25419228 PMC4240321

[wnan1546-bib-0001] Dobrovolskaia, M. A. , Aggarwal, P. , Hall, J. B. , & McNeil, S. E. (2008). Preclinical studies to understand nanoparticle interaction with the immune system and its potential effects on nanoparticle biodistribution. Molecular Pharmaceutics, 5(4), 487–495. 10.1021/mp800032f 18510338 PMC2613572

[wnan1546-bib-0002] Dobrovolskaia et al. (2008). Method for analysis of nanoparticle hemolytic properties in vitro. Nano Letters, 8(8), 2180–2187. 10.1021/nl0805615.Method 18605701 PMC2613576

[wnan1546-bib-0003] Fadeel, B. , N, F. , Vogt, C. , AM, A. , & WJ, P. (2013). Bridge over troubled waters: Understanding the synthetic and biological identities of engineered nanomaterials. Wiley Interdisciplinary Reviews: Nanomedicine and Nanobiotechnology, 5(2), 111–129. 10.1002/wnan.1206 23335558

[wnan1546-bib-0083] Aggarwal, P. , Hall, J. B. , McLeland, C. B. , Dobrovolskaia, M. A. , & McNeil, S. E. (2009). Nanoparticle interaction with plasma proteins as it relates to particle biodistribution, biocompatibility and therapeutic efficacy. Advanced Drug Delivery Reviews, 61(6), 428–437. 10.1016/j.addr.2009.03.009 19376175 PMC3683962

[wnan1546-bib-0006] ASTM E2524‐08 . (2013). Standard test method for analysis of hemolytic properties of nanoparticles.

[wnan1546-bib-0007] ASTM F 2382‐04 . (2004). Standard test method for assessment of intravascular medical device materials on partial thromboplastin time (PTT). 10.1520/F2382-04R10

[wnan1546-bib-0008] ASTM F1984‐99 . (2013). Standard practice for testing for whole complement activation in serum by solid materials. 10.1520/F1984-99R13

[wnan1546-bib-0009] ASTM F2888‐13 . (2013). Standard test method for platelet leukocyte count ‐ An in vitro measure for hemocompatibility assessment of cardiovascular materials.

[wnan1546-bib-0010] Braune, S. , Grunze, M. , Straub, A. , & Jung, F. (2013). Are there sufficient standards for the in vitro hemocompatibility testing of biomaterials? Biointerphases, 8(30), 33 10.1186/1559-4106-8-33 24706143

[wnan1546-bib-0011] Briggs, C. , Culp, N. , Davis, B. , d'Onofrio, G. , Zini, G. , & Machin, S. J. (2014). ICSH guidelines for the evaluation of blood cell analysers including those used for differential leucocyte and reticulocyte counting. International Journal of Laboratory Hematology, 36(6), 613–627. 10.1111/ijlh.12201 24666725

[wnan1546-bib-0012] Chanan‐Khan, A. , Szebeni, J. , Savay, S. , Liebes, L. , Rafique, N. M. , Alving, C. R. , & Muggia, F. M. (2003). Complement activation following first exposure to pegylated liposomal doxorubicin (Doxil®): Possible role in hypersensitivity reactions. Annals of Oncology, 14(9), 1430–1437. 10.1093/annonc/mdg374 12954584

[wnan1546-bib-0014] Choi, J. , Reipa, V. , Hitchins, V. M. , Goering, P. L. , & Malinauskas, R. A. (2011). Physicochemical characterization and in vitro hemolysis evaluation of silver nanoparticles. Toxicological Sciences, 123(1), 133–143. 10.1093/toxsci/kfr149 21652737

[wnan1546-bib-0015] CLSI . (1997). CLSI H48‐A: Determination of factor coagulant activities. Wayne, PA: Clinical and Laboratory Standards Institute.

[wnan1546-bib-0016] CLSI . (2001). CLSI H30‐A2:Procedure for the determination of fibrinogen in plasma. Approved guideline‐second edition. Wayne, PA: Clinical and Laboratory Standards Institute.

[wnan1546-bib-0017] CLSI . (2008a). CLSI H47‐A2: One‐stage prothrombin time (PT) test and activated partial thromboplastin time (APTT) test. Approved Guideline. Wayne, PA: Clinical and Laboratory Standards Institute.

[wnan1546-bib-0018] CLSI . (2008b). CLSI H58‐A: Platelet function testing by aggregometry. Wayne, PA: Clinical and Laboratory Standards Institute.

[wnan1546-bib-0022] Csaba, N. , Sánchez, A. , & Alonso, M. J. (2006). PLGA: Poloxamer and PLGA: Poloxamine blend nanostructures as carriers for nasal gene delivery. Journal of Controlled Release, 113(2), 164–172. 10.1016/j.jconrel.2006.03.017 16759732

[wnan1546-bib-0023] Curry, A. N. , & Pierce, T. (2007). Conventional and near‐patient tests of coagulation. Continuing Education in Anaesthesia Critical Care & Pain, 7(2), 45–50. 10.1093/bjaceaccp/mkm002

[wnan1546-bib-0024] Deng, Z. J. , Liang, M. , Monteiro, M. , Toth, I. , & Minchin, R. F. (2011). Nanoparticle‐induced unfolding of fibrinogen promotes Mac‐1 receptor activation and inflammation. Nature Nanotechnology, 6(1), 39–44. 10.1038/nnano.2010.250 21170037

[wnan1546-bib-0025] Deng, Z. J. , Liang, M. , Toth, I. , Monteiro, M. , & Minchin, R. F. (2013). Plasma protein binding of positively and negatively charged polymer‐coated gold nanoparticles elicits different biological responses. Nanotoxicology, 7(3), 314–322. 10.3109/17435390.2012.655342 22394123

[wnan1546-bib-0026] Desai, N. (2012). Challenges in development of nanoparticle‐based therapeutics. The AAPS Journal, 14(2), 282–295. 10.1208/s12248-012-9339-4 22407288 PMC3326161

[wnan1546-bib-0027] D'Mello, S. R. , Cruz, C. N. , Chen, M.‐L. , Kapoor, M. , Lee, S. L. , & Tyner, K. M. (2017). The evolving landscape of drug products containing nanomaterials in the United States. Nature Nanotechnology, 12(6), 523–529. 10.1038/nnano.2017.67 28436961

[wnan1546-bib-0084] Dobrovolskaia, M. A. , Shurin, M. , & Shvedova, A. A. (2016). Current understanding of interactions between nanoparticles and the immune system. Toxicology and Applied Pharmacology, 299(April), 78–89. 10.1016/j.taap.2015.12.022 26739622 PMC4811709

[wnan1546-bib-0028] Dobrovolskaia, M. A. , & McNeil, S. E. (2015). Strategy for selecting nanotechnology carriers to overcome immunological and hematological toxicities challenging clinical translation of nucleic acid‐based therapeutics. Expert Opinion on Drug Delivery, 12(7), 1163–1175. 10.1517/17425247.2015.1042857 25994601

[wnan1546-bib-0029] Dobrovolskaia, M. A. , & McNeil, S. E. (2013). Understanding the correlation between in vitro and in vivo immunotoxicity tests for nanomedicines. Journal of Controlled Release, 172(2), 456–466. 10.1016/j.jconrel.2013.05.025 23742883 PMC5831149

[wnan1546-bib-0085] Dobrovolskaia, M. A. , Neun, B. W. , Man, S. , Ye, X. , Hansen, M. , Patri, A. K. , … McNeil, S. E. (2014). Protein corona composition does not accurately predict hematocompatibility of colloidal gold nanoparticles. Nanomedicine: Nanotechnology, Biology, and Medicine, 10(7), 1453–1463. 10.1016/j.nano.2014.01.009 PMC4125554 24512761

[wnan1546-bib-0030] Dobrovolskaia, M. A. , Patri, A. K. , Simak, J. , Hall, J. B. , Semberova, J. , Lacerda, S. H. D. P. , & Mcneil, S. E. (2013). Nanoparticle size and surface charge determine effects of PAMAM dendrimers on human platelets in vitro. Molecular Pharmacology, 9(3), 382–393. 10.1021/mp200463e.Nanoparticle PMC3624701 22026635

[wnan1546-bib-0086] Dobrovolskaia, M. A. , Clogston, J. D. , Neun, B. W. , Hall, J. B. , Patri, A. K. , & McNeil, S. E. (2008). Method for analysis of nanoparticle hemolytic properties in vitro. Nano Letters, 8(8), 2180–2187. 18605701 10.1021/nl0805615 PMC2613576

[wnan1546-bib-0031] Domański, D. M. , Klajnert, B. , & Bryszewska, M. (2004). Influence of PAMAM dendrimers on human red blood cells. Bioelectrochemistry, 63(1–2), 189–191. 10.1016/j.bioelechem.2003.09.023 15110271

[wnan1546-bib-0032] European Clinical Trials Database (EUdraCT) . (2017). Retrieved from https://eudract.ema.europa.eu/index.html

[wnan1546-bib-0087] European Medicines Agency/Committee for Medicinal Products for Human Use . (2010). CPMP/SWP/1042/99 (R1)‐ Guideline on Repeated Dose Toxicity, 99 (March), 1–9.

[wnan1546-bib-0088] European Medicines Agency/Committee for Medicinal Products for Human Use . (2013a). Joint MHLW/EMA reflection paper on the development of block copolymer micelle medicinal products, EMA/CHMP/13099/2013.

[wnan1546-bib-0089] European Medicines Agency/Committee for Medicinal Products for Human Use . (2013b). Reflection paper on surface coatings: General issues for consideration regarding parenteral administration of coated nanomedicine products, EMA/325027/2013.

[wnan1546-bib-0090] European Medicines Agency/Committee for Medicinal Products for Human Use . (2013c). Reflection paper on the data requirements for intravenous liposomal products developed with reference to an innovator liposomal product, EMA/CHMP/806058/2009/Rev.02.

[wnan1546-bib-0034] Fadeel, B. (2013). Nanosafety: Towards safer design of nanomedicines. Journal of Internal Medicine, 274(6), 578–580. 10.1111/joim.12137 24102766

[wnan1546-bib-0035] Fadeel, B. , & Garcia‐Bennett, A. E. (2010). Better safe than sorry: Understanding the toxicological properties of inorganic nanoparticles manufactured for biomedical applications. Advanced Drug Delivery Reviews, 62(3), 362–374. 10.1016/j.addr.2009.11.008 19900497

[wnan1546-bib-0036] Giannakou, C. , Park, M. V. D. Z. , De Jong, W. H. , Van Loveren, H. , Vandebriel, R. J. , & Geertsma, R. E. (2016). A comparison of immunotoxic effects of nanomedicinal products with regulatory immunotoxicity testing requirements. International Journal of Nanomedicine, 11, 2935–2952. 10.2147/IJN.S102385 27382281 PMC4922791

[wnan1546-bib-0037] Guidetti, G. F. , Consonni, A. , Cipolla, L. , Mustarelli, P. , Balduini, C. , & Torti, M. (2012). Nanoparticles induce platelet activation in vitro through stimulation of canonical signalling pathways. Nanomedicine, 8, 1329–1336. 10.1016/j.nano.2012.04.001 22542822

[wnan1546-bib-0038] Guildford, A. L. , Poletti, T. , Osbourne, L. H. , Di Cerbo, A. , Gatti, A. M. , & Santin, M. (2009). Nanoparticles of a different source induce different patterns of activation in key biochemical and cellular components of the host response. Journal of the Royal Society Interface, 6(41), 1213–1221. 10.1098/rsif.2009.0021 PMC2817157 19324665

[wnan1546-bib-0091] Halamoda‐Kenzaoui, B. , & Bremer‐Hoffmann, S. (2018a). Main trends of immune effects triggered by nanomedicines in preclinical studies. International Journal of Nanomedicine, 13, 5419–5431. 10.2147/IJN.S168808 30271138 PMC6149906

[wnan1546-bib-0092] Halamoda‐Kenzaoui, B. , Holzwarth, U. , Roebben, G. , Bogni, A. , & Bremer‐Hoffmann, S. (2018b). Mapping of the available standards against the regulatory needs for nanomedicines. WIREs Nanomedine and Nanobiotechnology, e1531 10.1002/wnan.1531 PMC6585614 29923692

[wnan1546-bib-0039] Hall, J. B. , Dobrovolskaia, M. A. , Patri, A. K. , & McNeil, S. E. (2007). Characterization of nanoparticles for therapeutics. Nanomedicine, 2(6), 789–803. 10.2217/17435889.2.6.789 18095846

[wnan1546-bib-0040] Hernán Pérez de la Ossa, D. (2014). Presentation: Quality aspects of nano‐based medicines. SME Workshop for micro, small and medium‐sized enterprises: Focus on quality for medicines containing chemical entities. Retrieved from https://www.ema.europa.eu/documents/presentation/presentation-quality-aspects-nano-based-medicines-dolores-hernan-pacrez-de-la-ossa_en.pdf

[wnan1546-bib-0041] Huang, H. , Lai, W. , Cui, M. , Liang, L. , Lin, Y. , Fang, Q. , … Xie, L. (2016). An evaluation of blood compatibility of silver nanoparticles. Scientific Reports, 6(April), 25518 10.1038/srep25518 27145858 PMC4857076

[wnan1546-bib-0042] ICH . (2005). ICH S8 ‐ Immunotoxicity studies for human pharmaceuticals, (September).

[wnan1546-bib-0043] ICH . (2009). ICH guideline M3(R2) on non‐clinical safety studies for the conduct of human clinical trials and marketing authorisation for pharmaceuticals. International Conference of Harmonisation of Technical Requirements for Registration of Pharmaceuticals for Human Use, 3(June). Retrieved from https://doi.org/EMA/CPMP/ICH/286/1995

[wnan1546-bib-0044] Ilinskaya, A. N. , & Dobrovolskaia, M. A. (2013). Nanoparticles and the blood coagulation system. Part II: Safety concerns. Nanomedicine, 8(5), 773–784. 10.2217/nnm.13.48 23730696 PMC3939602

[wnan1546-bib-0045] ISO . (2017a). ISO /TR 10993‐22. Biological evaluation of medical devices‐ Part 22: Guidance on nanomaterials.

[wnan1546-bib-0046] ISO . (2017b). ISO 10993‐4 : Biological evaluation of medical devices. Part 4: Selection of tests for interaction with blood.

[wnan1546-bib-0047] Kaplan, W. , Wirtz, V. , Mantel‐Teeuwisse, A. , Stolk, P. , Duthey, B. , & Laining, R. (2013). Priority Medicines for Europe and the World. 2013 Update. World Health Organization in Collaboration with Utrecht University and Boston University.

[wnan1546-bib-0048] Kaur, I. P. , Kakkar, V. , Deol, P. K. , Yadav, M. , Singh, M. , & Sharma, I. (2014). Issues and concerns in nanotech product development and its commercialization. Journal of Controlled Release, 193(2014), 51–62. 10.1016/j.jconrel.2014.06.005 24933600

[wnan1546-bib-0049] Klee, G. , D'Onofrio, G. , Van Assendelft, O. W. , Bull, B. , Bunyaratvej, A. , Buttarello, M. , … Lakomsky, D. (2001). Platelet counting by the RBC/platelet ratio method: A reference method. American Journal of Clinical Pathology, 115(3), 460–464. 10.1309/W612-MYEP-FA7U-8UYA 11246942

[wnan1546-bib-0050] Kola, I. , & Landis, J. (2004). Can the pharmaceutical industry reduce attrition rates? Nature Reviews Drug Discovery, 3, 711–716. 10.1038/nrd1470 15286737

[wnan1546-bib-0051] Kratz, A. , Plebani, M. , Peng, M. , Lee, Y. K. , McCafferty, R. , & Machin, S. J. (2017). ICSH recommendations for modified and alternate methods measuring the erythrocyte sedimentation rate. International Journal of Laboratory Hematology, *39*(5), 448–457. 10.1111/ijlh.12693 28497537

[wnan1546-bib-0052] Laloy, J. , Minet, V. , Alpan, L. , Mullier, F. , Beken, S. , Toussaint, O. , … Dogné, J.‐M. (2014). Impact of silver nanoparticles on haemolysis, platelet function and coagulation. Nanobiomedicine, 1, 1–9. 10.5772/59346 30023015 PMC6029236

[wnan1546-bib-0053] Laloy, J. , Robert, S. , Marbehant, C. , Mullier, F. , Mejia, J. , Piret, J.‐P. , … Rolin, S. (2012). Validation of the calibrated thrombin generation test (cTGT) as the reference assay to evaluate the procoagulant activity of nanomaterials. Nanotoxicology, 6(2), 213–232. 10.3109/17435390.2011.569096 21486188

[wnan1546-bib-0093] Lee, Y. K. , Choi, E. J. , Webster, T. J. , Kim, S. H. , & Khang, D. (2014). Effect of the protein corona on nanoparticles for modulating cytotoxicity and immunotoxicity. International Journal of Nanomedicine, 10, 97–113. 10.2147/IJN.S72998 25565807 PMC4275058

[wnan1546-bib-0054] Li, S. Q. , Zhu, R. R. , Zhu, H. , Xue, M. , Sun, X. Y. , De Yao, S. , & Wang, S. L. (2008). Nanotoxicity of TiO2 nanoparticles to erythrocyte in vitro. Food and Chemical Toxicology, 46(12), 3626–3631. 10.1016/j.fct.2008.09.012 18840495

[wnan1546-bib-0055] Lundqvist, M. , Augustsson, C. , Lilja, M. , Lundkvist, K. , Dahlbäck, B. , Linse, S. , & Cedervall, T. (2017). The nanoparticle protein corona formed in human blood or human blood fractions. PLoS One, 12(4), 1–15. 10.1371/journal.pone.0175871 PMC5393619 28414772

[wnan1546-bib-0056] Malik, N. , Wiwattanapatapee, R. , Klopsch, R. , Lorenz, K. , Frey, H. , Weener, J. W. , … Duncan, R. (2000). Dendrimers: Relationship between structure and biocompatibility in vitro, and preliminary studies on the biodistribution of 125I‐labelled polyamidoamine dendrimers in vivo. Journal of Controlled Release, 65(1–2), 133–148. 10.1016/S0168-3659(99)00246-1 10699277

[wnan1546-bib-0057] Markiewski, M. M. , Nilsson, B. , Nilsson Ekdahl, K. , Mollnes, T. E. , & Lambris, J. D. (2007). Complement and coagulation: Strangers or partners in crime? Trends in Immunology, 28(4), 184–192. 10.1016/j.it.2007.02.006 17336159

[wnan1546-bib-0058] Mayer, A. , Vadon, M. , Rinner, B. , Novak, A. , Wintersteiger, R. , & Fröhlich, E. (2009). The role of nanoparticle size in hemocompatibility. Toxicology, 258(2–3), 139–147. 10.1016/j.tox.2009.01.015 19428933

[wnan1546-bib-0059] Moghimi, S. M. , & Hamad, I. (2010). Hypersensitivity reactions to nanomedicines: Causative factors and optimization of design parameters In R. Pawankar, S.T. Holgate & L. J. Rosenwasser (Eds.), Allergy Frontiers: Future Perspectives. Allergy Frontiers (Vol. 6, pp.225–237). Tokyo, Japan: Springer.

[wnan1546-bib-0060] Nel, A. E. (2013). Implementation of alternative test strategies for the safety assessment of engineered nanomaterials. Journal of Internal Medicine, 274(6), 561–577. 10.1111/joim.12109 23879741 PMC4096910

[wnan1546-bib-0061] Onakpoya, I. J. , Heneghan, C. J. , & Aronson, J. K. (2016). Post‐marketing withdrawal of 462 medicinal products because of adverse drug reactions: A systematic review of the world literature. BMC Medicine, 14(1), 10 10.1186/s12916-016-0553-2 26843061 PMC4740994

[wnan1546-bib-0062] Quiros‐Pesudo, L. , Balahur‐Dobrescu, A. , Gottardo, S. , Rasmussen, K. , Wagner, G. , Joanny, G. , & Bremer, S. (2018). Mapping nanomedicine terminology in the regulatory landscape. Luxembourg: Publications Office of the European. 10.2760/753829

[wnan1546-bib-0063] Radomski, A. , Jurasz, P. , Alonso‐Escolano, D. , Drews, M. , Morandi, M. , Malinski, T. , & Radomski, M. W. (2005). Nanoparticle‐induced platelet aggregation and vascular thrombosis. British Journal of Pharmacology, 146(6), 882–893. 10.1038/sj.bjp.0706386 16158070 PMC1751219

[wnan1546-bib-0064] Reddy, S. T. , Van der Vlies, A. J. , Simeoni, E. , O'Neil, C. P. , Swartz, M. A. , & Hubbell, J. A. (2007). Exploiting lymphatic transport and complement activation in nanoparticle vaccines. Nature Biotechnology, 25(10), 1159–1164. 10.1038/nbt1332 17873867

[wnan1546-bib-0065] Grand View Research . (2017). Nanomedicine market analysis by products, (therapeutics, regenerative medicine, diagnostics), by application, (clinical oncology, infectious diseases), by nanomolecule (gold, silver, iron oxide, alumina)*,* & segment forecasts, 2013–2025. Retrived from https://doi.org/978-1-68038-942-5

[wnan1546-bib-0066] Roussel, M. , Davis, B. H. , Fest, T. , & Wood, B. L. (2012). Toward a reference method for leukocyte differential counts in blood: Comparison of three flow cytometric candidate methods. Cytometry Part A, 81 A(11), 973–982. 10.1002/cyto.a.22092 22736499

[wnan1546-bib-0067] Sanfins, E. , Augustsson, C. , Dahlbäck, B. , Linse, S. , & Cedervall, T. (2014). Size‐dependent effects of nanoparticles on enzymes in the blood coagulation cascade. Nano Letters, 14(8), 4736–4744. 10.1021/nl501863u 25025946

[wnan1546-bib-0094] Setyawati, M. I. , Tay, C. Y. , Docter, D. , Stauber, R. H. , & Leong, D. T. (2015). Understanding and exploiting nanoparticles’ intimacy with the blood vessel and blood. Chemical Society Reviews, 44(22), 8174–8199. 10.1039/c5cs00499c 26239875

[wnan1546-bib-0068] Shrivastava, S. , Bera, T. , Singh, S. K. , Singh, G. , Ramachandrarao, P. , & Dash, D. (2009). Characterization of antiplatelet properties of silver nanoparticles. ACS Nano, 3(6), 1357–1364. 10.1021/nn900277t 19545167

[wnan1546-bib-0069] Szebeni, J. (2012). Hemocompatibility testing for nanomedicines and biologicals: Predictive assays for complement mediated infusion reactions. European Journal of Nanomedicine, 4(1), 33–53. 10.1515/ejnm-2012-0002

[wnan1546-bib-0070] Szebeni, J. , Muggia, F. , Gabizon, A. , & Barenholz, Y. (2011). Activation of complement by therapeutic liposomes and other lipid excipient‐based therapeutic products: Prediction and prevention. Advanced Drug Delivery Reviews, 63(12), 1020–1030. 10.1016/j.addr.2011.06.017 21787819

[wnan1546-bib-0071] Szebeni, J. , & Storm, G. (2015). Complement activation as a bioequivalence issue relevant to the development of generic liposomes and other nanoparticulate drugs. Biochemical and Biophysical Research Communications, 468(3), 490–497. 10.1016/j.bbrc.2015.06.177 26182876

[wnan1546-bib-0072] Thevenot, P. , Hu, W. , & Tang, L. (2008). Surface chemistry influence implant biocompatibility. Current Topics in Medicinal Chemistry, 8(4), 270–280. 18393890 10.2174/156802608783790901 PMC3230929

[wnan1546-bib-0073] US FDA Center for Drug Evaluation and Research (CDER); Center for Biologics Evaluation and Research (CBER) . (2017). Guidance on drug products, including biological products, that contain nanomaterials ‐ guidance for industry, (December). Retrieved from https://www.fda.gov/Drugs/GuidanceComplianceRegulatoryInformation/Guidances/default.htm

[wnan1546-bib-0074] Van Oeveren, W. (2013). Obstacles in Haemocompatibility testing. Scientifica, 2013, 1–14. 10.1155/2013/392584 PMC3820147 24278774

[wnan1546-bib-0075] Vauthier, C. , Persson, B. , Lindner, P. , & Cabane, B. (2011). Protein adsorption and complement activation for di‐block copolymer nanoparticles. Biomaterials, 32(6), 1646–1656. 10.1016/j.biomaterials.2010.10.026 21093043

[wnan1546-bib-0076] Vonarbourg, A. , Passirani, C. , Saulnier, P. , Simard, P. , Leroux, J. C. , & Benoit, J. P. (2006). Evaluation of pegylated lipid nanocapsules versus complement system activation and macrophage uptake. Clinical and Experimental Rheumatology, 78A(3), 620–628. 10.1002/jbm.a 16779767

[wnan1546-bib-0077] Wang, G. , Chen, F. , Banda, N. K. , Holers, V. M. , Wu, L. P. , Moghimi, S. M. , & Simberg, D. (2016). Activation of human complement system by dextran‐coated iron oxide nanoparticles is not affected by dextran/Fe ratio, hydroxyl modifications, and crosslinking. Frontiers in Immunology, 7(OCT), 1–8. 10.3389/fimmu.2016.00418 27777575 PMC5056169

[wnan1546-bib-0095] Weingand, K. , Brown, G. , Hall, R. , Davies, D. , Gossett, K. , Neptun, D. , … Melloni, E. (1996). Harmonization of animal clinical pathology testing in toxicity and safety studies. Fundamental and Applied Toxicology, 29(2), 198–201. 10.1093/toxsci/29.2.198 8742316

[wnan1546-bib-0078] Wibroe, P. P. , & Moghimi, S. M. (2012). Complement sensing of nanoparticles and nanomedicines In Functional nanoparticles for bioanalysis, nanomedicine, and bioelectronic devices (Vol. 2, pp. 365–382). Washington, DC: American Chemical Society. 10.1021/bk-2012-1113.ch014

[wnan1546-bib-0079] Wicki, A. , Witzigmann, D. , Balasubramanian, V. , & Huwyler, J. (2015). Nanomedicine in cancer therapy: Challenges, opportunities, and clinical applications. Journal of Controlled Release, 200, 138–157. 10.1016/j.jconrel.2014.12.030 25545217

[wnan1546-bib-0080] Zhao, Y. , Sun, X. , Zhang, G. , Trewyn, B. G. , Slowing, I. I. , & Lin, V. S.‐Y. (2011). Interaction of mesoporous silica nanoparticles with human red blood cell membranes : Size and surface effects. ACS Nano, 5(2), 1366–1375. 10.1021/nn103077k 21294526

