# Supplementary material for: Overview of the blood compatibility of nanomedicines: A trend analysis of in vitro and in vivo studies
Source: Wiley Interdiscip Rev Nanomed Nanobiotechnol. 2018 Dec 17;11(3):e1546. doi: 10.1002/wnan.1546 (PMC7816241; doi:10.1002/wnan.1546)
Supplement: Supplementary file 1 — Table S1. List of publications used for in vivo analysis. Information regarding the test category, the category of material, the type of material and the reference to the article is shown. [file WNAN-11-e1546-s001.docx]

| **Test Category** | **Category of material** | **Type of material** | **Authors** | **Journal** | **Year** |
| --- | --- | --- | --- | --- | --- |
| Haematology | Inorganic | Gold NPs | Abdelhalim et al | Journal of Nanomedicine and Nanotechnology | 2012 |
| Haematology | Polymer-based | Polymers | Anitha et al | Biochimica et Biophysica Acta | 2014 |
| Haematology | Polymer-based | Polymers | Anitha et al | European Journal of Pharmaceutics and Biopharmaceutics | 2014 |
| Haematology | Inorganic | Gold NPs | Bucharskaya et al | Journal of Innovative Optical Health Sciences | 2016 |
| Haematology | Lipid-based | Liposomes | Carvalheiro et al | European Journal of Pharmaceutics and Biopharmaceutics | 2015 |
| Haematology | Inorganic | Gold NPs | Chanda et al | Nanomedicine: Nanotechnology, Biology, and Medicine | 2010 |
| Haematology | Polymer-based | Dendrimers | Chen et al | Journal of the American Chemical Society | 2004 |
| Haematology | Inorganic | Iron oxide NPs | Couto et al | Journal of Applied Toxicology | 2016 |
| Haematology | Polymer-based | Micelles | Gong et al | Journal of Nanoparticle Research | 2011 |
| Haematology | Polymer-based | Micelles | Gou et al | Pharmaceutical Research | 2009 |
| Haematology | Polymer-based | Polymers | Hamilton et al | Biomaterials | 2016 |
| Haematology | Lipid-based | Niosomes | Imran et al | Drug Delivery | 2016 |
| Haematology | Polymer-based | Dendrimers | Imran Ul-Haq et al | ACS Nano | 2013 |
| Haematology | Lipid-based | Nanoemulsion | Jing et al | Nanomedicine: Nanotechnology, Biology, and Medicine | 2014 |
| Haematology | Polymer-based | Dendrimers | Kesharwani et al | Nanomedicine | 2014 |
| Haematology | Polymer-based | Polymers | Kumar et al | Journal of Biomaterials and Tissue Engineering | 2014 |
| Haematology | Inorganic | Nanocrystals | Li et al | Scientific Reports | 2013 |
| Haematology | Polymer-based | Dendrimers | Liang et al | International Journal of Nanomedicine | 2014 |
| Haematology | Inorganic | Silica NPs | Liu et al | Drug Development and Industrial Pharmacy | 2016 |
| Haematology | Polymer-based | Polymeric NPs | Liu et al | Biomaterials | 2013 |
| Haematology | Lipid-based | Liposomes | Lopes et al | European Journal of Pharmaceutics and Biopharmaceutics | 2012 |
| Haematology | Inorganic | Titanium dioxide NPs | Lucky et al | ACS Nano | 2015 |
| Haematology | Lipid-based | Micelles | Ma et al | Journal of Controlled Release | 2015 |
| Haematology | Lipid-based | Nanoemulsion | Mahmoud et al | International Journal of Pharmaceutics | 2014 |
| Haematology | Polymer-based | Polymeric NPs | Maya et al | Journal of Biomedical Nanotechnology | 2014 |
| Haematology | Lipid-based | Nanoemulsion | Melariri et al | International Journal of Nanomedicine | 2015 |
| Haematology | Polymer-based | Dendrimers | Mishra et al | International Journal of Pharmaceutics | 2014 |
| Haematology | Polymer-based | Polymers | Narayanan et al | Acta Biomaterialia | 2014 |
| Haematology | Lipid-based | Micelles | Oberoi et al | International Journal of Nanomedicine | 2012 |
| Haematology | Inorganic | Nanocrystals | Pawar et al | Journal of Biomedical Nanotechnology | 2015 |
| Haematology | Inorganic | Gold NPs | Sengupta et al | Journal of Nanoscience and Nanotechnology | 2013 |
| Haematology | Polymer-based | Dendrimers | Swami et al | Journal of Nanoparticle Research | 2015 |
| Haematology | Polymer-based | Polymeric NPs | Urbán et al | Journal of Controlled Release | 2014 |
| Haematology | Inorganic | Iron oxide NPs | Wang et al | World Journal of Gastroenterology | 2009 |
| Haematology | Polymer-based | Dendrimers | Xie et al | International Journal of Pharmaceutics | 2015 |
| Haematology | Polymer-based | Polymeric NPs | Xu et al | Molecular Pharmaceutics | 2016 |
| Haematology | Polymer-based | Polymeric NPs | Yang et al | International Journal of Nanomedicine | 2012 |
| Haematology | Lipid-based | Liposomes | Yang et al | International Journal of Pharmaceutics | 2015 |
| Haematology | Inorganic | Silica NPs | Zhang et al | Nanoscale | 2015 |
| Haematology | Inorganic | Gold NPs | Zhang et al | International Journal of Nanomedicine | 2010 |
| Haematology | Polymer-based | Polymeric NPs | Zhou et al | Anticancer Research | 2016 |
| Coagulation | Inorganic | Gold NPs | Abdelhalim et al | Journal of Nanomedicine and Nanotechnology | 2012 |
| Coagulation | Polymer-based | Polymeric NPs | Anselmo et al | ACS Nano | 2014 |
| Coagulation | Polymer-based | Polymers | Avery et al | Science Translational Medicine | 2016 |
| Coagulation | Polymer-based | Polymeric NPs | Bagre et al | International journal of pharmaceutics | 2013 |
| Coagulation | Inorganic | Carbon nanotubes | Bihari et al | Toxicology | 2010 |
| Coagulation | Inorganic | Carbon nanotubes | Burke et al | Biomaterials | 2011 |
| Coagulation | Lipid-based | Micelles | Chan et al | Biomaterials | 2016 |
| Coagulation | Inorganic | Gold NPs | Chen et al | ACS Applied Materials and Interfaces | 2016 |
| Coagulation | Inorganic | Titanium dioxide NPs | Chen et al | Journal of Applied Toxicology | 2009 |
| Coagulation | Inorganic | Iron oxide NPs | Chen et al | Int J Nanomedicine | 2016 |
| Coagulation | Polymer-based | Polymers | De Valence et al | Acta Biomaterialia | 2012 |
| Coagulation | Polymer-based | Polymers | De Valence et al | Biomaterials | 2012 |
| Coagulation | Inorganic | Carbon nanotubes | Gaffney et al | Nanomedicine: Nanotechnology, Biology, and Medicine | 2015 |
| Coagulation | Lipid-based | Liposomes | Ganly et al | Journal of Controlled Release | 2013 |
| Coagulation | Inorganic | Quantum dots | Geys et al | Environmental Health Perspectives | 2008 |
| Coagulation | Inorganic | Titanium dioxide NPs | Haberl et al | Nanotoxicology | 2015 |
| Coagulation | Inorganic | Silica NPs | Haberl et al | Nanotoxicology | 2015 |
| Coagulation | Inorganic | Silver NPs | Haberl et al | Nanotoxicology | 2015 |
| Coagulation | Inorganic | Iron oxide NPs | Herrmann et al | Nanomedicine | 2011 |
| Coagulation | Inorganic | Carbon nanotubes | Holzer et al | Journal of Applied Toxicology | 2014 |
| Coagulation | Inorganic | Silica NPs | Hudson et al | Biomaterials | 2008 |
| Coagulation | Polymer-based | Polymeric NPs | Ignjatović et al | Materials Science and Engineering C | 2016 |
| Coagulation | Polymer-based | Dendrimers | Imran ul-haq | ACS Nano | 2013 |
| Coagulation | Inorganic | Iron oxide NPs | Israel et al | ACS Applied Materials and Interfaces | 2015 |
| Coagulation | Polymer-based | Polymers | Jia et al | Journal of Biomedical Materials Research - Part A | 2011 |
| Coagulation | Polymer-based | Polymers | Jiang et al | Drug Design, Development and Therapy | 2016 |
| Coagulation | Polymer-based | Polymers | Jogala et al | International Journal of Pharmacy and Pharmaceutical Sciences | 2016 |
| Coagulation | Polymer-based | Polymers | Jogala et al | Current Drug Delivery | 2016 |
| Coagulation | Polymer-based | Dendrimers | Jones et al | ACS Nano | 2012 |
| Coagulation | Inorganic | Silver NPs | Jun et al | Nanotoxicology | 2011 |
| Coagulation | Polymer-based | Polymers | Karagkiozaki et al | Biochimica et Biophysica Acta | 2013 |
| Coagulation | Inorganic | Carbon nanotubes | Karagkiozaki et al | International Journal of Nanomedicine | 2012 |
| Coagulation | Inorganic | Iron oxide NPs | Kempe et al | Biomaterials | 2010 |
| Coagulation | Polymer-based | Polymeric NPs | Kim et al | Journal of Agricultural and Food Chemistry | 2016 |
| Coagulation | Polymer-based | Polymers | Koppara et al | International Journal of Cardiology | 2016 |
| Coagulation | Inorganic | Nanodiamonds | Kumari et al | Nanomedicine | 2014 |
| Coagulation | Polymer-based | Polymers | Lai et al | Biomaterials | 2010 |
| Coagulation | Polymer-based | Polymers | Lee et al | International Journal of Nanomedicine | 2014 |
| Coagulation | Inorganic | Nanocrystals | Li et al | Scientific Reports | 2013 |
| Coagulation | Polymer-based | Polymers | Li et al | Nanomedicine: Nanotechnology, Biology, and Medicine | 2011 |
| Coagulation | Polymer-based | Polymers | Liu et al | Journal of Clinical Rehabilitative Tissue Engineering Research | 2008 |
| Coagulation | Polymer-based | Polymers | Liu et al | ACS Applied Materials and Interfaces | 2014 |
| Coagulation | Polymer-based | Polymers | Ma et al | Journal of Clinical Rehabilitative Tissue Engineering Research | 2008 |
| Coagulation | Polymer-based | Polymers | Major et al | Biomaterials | 2011 |
| Coagulation | Polymer-based | Polymeric NPs | Masuda et al | Journal of Atherosclerosis and Thrombosis | 2011 |
| Coagulation | Polymer-based | Polymeric NPs | Mei et al | Chinese Science Bulletin | 2010 |
| Coagulation | Lipid-based | Lipid NPs | Metzger et al | Thrombosis and Haemostasis | 2015 |
| Coagulation | Polymer-based | Polymeric NPs | Morey et al | Journal of Nanoparticle Research | 2004 |
| Coagulation | Inorganic | Titanium dioxide NPs | Nemmar et al | Experimental Biology and Medicine | 2008 |
| Coagulation | Inorganic | Silica nanoparticles | Nemmar et al | International Journal of Nanomedicine | 2014 |
| Coagulation | Inorganic | Iron oxide NPs | Nemmar et al | Particle and Fibre Toxicology | 2016 |
| Coagulation | Lipid-based | Liposomes | Okamura et al | Journal of Thrombosis and Haemostasis | 2009 |
| Coagulation | Inorganic | Perfluorocarbon NPs | Palekar et al | Journal of Vascular Surgery | 2016 |
| Coagulation | Polymer-based | Polymeric NPs | Paliwal et al | International Journal of Pharmaceutics | 2012 |
| Coagulation | Polymer-based | Polymeric NPs | Pazzini et al | Journal of Nanoscience and Nanotechnology | 2015 |
| Coagulation | Inorganic | Carbon nanotubes | Radomski et al | British Journal of Pharmacology | 2005 |
| Coagulation | Polymer-based | Polymer | Sarkar et al | Journal of Biomechanics | 2009 |
| Coagulation | Inorganic | Gold NPs | Shah et al | BioNanoMaterials | 2013 |
| Coagulation | Lipid-based | Micelles | She et al | ACS Nano | 2014 |
| Coagulation | Polymer-based | Polymeric NPs | Shi et al | Biomaterials | 2012 |
| Coagulation | Inorganic | Silver NPs | Shrivastava et al | ACS Nano | 2009 |
| Coagulation | Inorganic | Iron oxide NPs | Simberg et al | Journal of Controlled Release | 2009 |
| Coagulation | Inorganic | Graphene oxide | Singh et al | ACS Nano | 2012 |
| Coagulation | Inorganic | Graphene oxide | Singh et al | ACS Nano | 2011 |
| Coagulation | Inorganic | Polystyrene NPs | Smyth et al | Nanotoxicology | 2015 |
| Coagulation | Polymer-based | Polymer | Solouk et al | Materials Science and Engineering C | 2015 |
| Coagulation | Inorganic | Iron oxide NPs | Starmans et al | PLoS ONE | 2015 |
| Coagulation | Inorganic | Iron oxide NPs | Suzuki et al | Nanomedicine | 2015 |
| Coagulation | Inorganic | Graphene oxide | Syama et al | International Journal of Biological Macromolecules | 2016 |
| Coagulation | Inorganic | Iron oxide NPs | Szekeres et al | International Journal of Molecular Sciences | 2013 |
| Coagulation | Inorganic | Carbon nanocapsules | Tang et al | ACS Nano | 2012 |
| Coagulation | Inorganic | Iron oxide NPs | Ternent et al | Journal of Materials Chemistry B | 2016 |
| Coagulation | Inorganic | Silver NPs | Tiwari et al | Toxicology Mechanisms and Methods | 2011 |
| Coagulation | Polymer-based | Polymer | Tseng et al | Journal of Biomedical Materials Research. Part A | 2011 |
| Coagulation | Inorganic | Iron oxide NPs | Tombácz et al | Interface Focus | 2016 |
| Coagulation | Inorganic | Hydroxyapatite NPs | Von Stechow et al | European Surgical Research | 2009 |
| Coagulation | Inorganic | Iron oxide NPs | Wuang et al | Advanced Functional Materials | 2006 |
| Coagulation | Inorganic | Graphene oxide | Xu et al | ACS Nano | 2016 |
| Coagulation | Lipid-based | Lipid NPs | Xu et al | Journal of Huazhong University of Science and Technology - Medical Science | 2013 |
| Coagulation | Lipid-based | Lipid NPs | Zhao et al | Journal of Interventional Radiology (China) | 2012 |
| Coagulation | Polymer-based | Polymeric NPs | Zheng et al | Journal of Biomedical Nanotechnology | 2014 |
| Complement | Lipid-based | Liposomes | Bugna et al | Nanomedicine: Nanotechnology, Biology, and Medicine | 2016 |
| Complement | Polymer-based | Polymeric NPs | D'Addio et al | Journal of Controlled Release | 2012 |
| Complement | Polymer-based | Polymeric NPs | De Souza et al | Nanotechnology | 2015 |
| Complement | Lipid-based | Liposomes | Dézsi et al | Journal of Controlled Release | 2014 |
| Complement | Inorganic | Carbon nanotubes | Hamad et al | Molecular Immunology | 2008 |
| Complement | Inorganic | Titanium dioxide NPs | Husain et al | Nanotoxicology | 2015 |
| Complement | Polymer-based | Dendrimers | Imran ul-haq M | ACS Nano | 2013 |
| Complement | Inorganic | Iron oxide NPs | Inturi et al | ACS Nano | 2015 |
| Complement | Polymer-based | Polymers | La Flamme et al | Biomaterials | 2007 |
| Complement | Inorganic | Silver NPs | Meng et al | Nanotoxicology | 2014 |
| Complement | Lipid-based | Liposomes | Mészáros et al | European Journal of Nanomedicine | 2015 |
| Complement | Polymer-based | Polymeric NPs | Naahidi et al | Molecular Pharmaceutics | 2014 |
| Complement | Polymer-based | Polymeric NPs | Passirani et al | Life Sciences | 1998 |
| Complement | Lipid-based | Lipid NPs | Pham et al | Journal of Biological Chemistry | 2011 |
| Complement | Inorganic | Perfluorocarbon NPs | Pham et al | Nanomedicine: Nanotechnology, Biology, and Medicine | 2014 |
| Complement | Polymer-based | Polymeric NPs | Reddy et al | European Cells and Materials | 2007 |
| Complement | Polymer-based | Polymeric NPs | Robbins et al | Nanomedicine: Nanotechnology, Biology, and Medicine | 2015 |
| Complement | Polymer-based | Polymeric NPs | Shan et al | Biomedical Microdevices | 2009 |
| Complement | Lipid-based | Liposomes | Szebeni et al | Journal of Controlled Release | 2012 |
| Complement | Lipid-based | Liposomes | Szebeni et al | Nanomedicine: Nanotechnology, Biology, and Medicine | 2012 |
| Complement | Inorganic | Iron oxide NPs | Wang et al | ACS Nano | 2014 |
| Complement | Inorganic | Iron oxide NPs | Wang et al | Scientific Reports | 2015 |
| Complement | Lipid-based | Liposomes | Yang et al | International Journal of Nanomedicine | 2013 |
| Complement | Inorganic | Gold NPs | You et al | Particle and Fibre Toxicology | 2014 |
