# Supplementary material for: Overview of the blood compatibility of nanomedicines: A trend analysis of in vitro and in vivo studies
Source: Wiley Interdiscip Rev Nanomed Nanobiotechnol. 2018 Dec 17;11(3):e1546. doi: 10.1002/wnan.1546 (PMC7816241; doi:10.1002/wnan.1546)
Supplement: Supplementary file 2 — Table S2. List of publications used for in vitro analysis. Information regarding the test category, the category of material, the type of material and the reference to the article is shown. [file WNAN-11-e1546-s002.docx]

| **Test Category** | **Category of material** | **Type of material** | **Authors** | **Journal** | **Year** |
| --- | --- | --- | --- | --- | --- |
| Haematology | Polymer-based | Polymeric NPs | Agashe et al | Journal of Pharmacy and Pharmacology | 2006 |
| Haematology | Inorganic | Titanium dioxide | Aich et al | Materials Letters | 2016 |
| Haematology | Polymer-based | Dendrimers | Alavidjeh et al | Journal of Materials Science: Materials in Medicine | 2010 |
| Haematology | Inorganic | Iron oxide | Almaki et al | Nanotechnology | 2016 |
| Haematology | Polymer-based | Polymeric NPs | Angelova et al | Colloids and Surfaces A: Physicochemical and Engineering Aspects | 2017 |
| Haematology | Polymer-based | Polymeric NPs | Angelova et al | Colloids and Surfaces A: Physicochemical and Engineering Aspects | 2015 |
| Haematology | Polymer-based | Polymeric NPs | Anitha et al | Journal of Biomedical Nanotechnology | 2012 |
| Haematology | Polymer-based | Polymeric NPs | Anitha et al | Biochimica et Biophysica Acta - General Subjects | 2014 |
| Haematology | Polymer-based | Polymeric NPs | Anitha et al | Journal of Biomaterials Science, Polymer Edition | 2012 |
| Haematology | Polymer-based | Polymeric NPs | Anitha et al | European Journal of Pharmaceutics and Biopharmaceutics | 2014 |
| Haematology | Inorganic | Iron oxide | Ansari et al | ACS Biomaterials Science and Engineering | 2016 |
| Haematology | Polymer-based | Nanogels | Arunraj et al | Colloids and Surfaces B: Biointerfaces | 2014 |
| Haematology | Inorganic | Zinc oxide NPs | Aula et al | Materials Research Express | 2014 |
| Haematology | Inorganic | Lutetium hydroxycarbonate NPs | Ba et al | New Journal of Chemistry | 2015 |
| Haematology | Polymer-based | Polymeric NPs | Barshtein et al | IEEE Transactions on Nanobioscience | 2011 |
| Haematology | Inorganic | Nanocrystals | Beeran et al | Colloids and Surfaces B: Biointerfaces | 2015 |
| Haematology | Polymer-based | Polymers | Bera et al | Journal of Materials Chemistry B | 2015 |
| Haematology | Inorganic | Iron oxide NPs | Bhattacharya et al | Materials Science and Engineering C | 2014 |
| Haematology | Inorganic | Iron oxide NPs | Bomati-Miguel et al | Journal of Nanoparticle Research | 2014 |
| Haematology | Polymer-based | Polymeric NPs | Brambilla et al | ACS Nano | 2012 |
| Haematology | Inorganic | Carbon nanotubes | Bussy et al | Advanced Drug Delivery Reviews | 2013 |
| Haematology | Inorganic | Graphene oxide | Cai et al | Applied Surface Science | 2015 |
| Haematology | Polymer-based | Polymeric NPs | Cavalli et al | Colloids and Surfaces B: Biointerfaces | 2015 |
| Haematology | Polymer-based | Polymeric NPs | Cavalli et al | International Journal of Nanomedicine | 2012 |
| Haematology | Inorganic | Gold NPs | Centi et al | Journal of Nanobiotechnology | 2014 |
| Haematology | Inorganic | Titanium oxide NPs | Chakrabartty et al | Journal of Nanoscience and Nanotechnology | 2016 |
| Haematology | Inorganic | Gold NPs | Chanda et al | Nanomedicine: Nanotechnology, Biology, and Medicine | 2010 |
| Haematology | Polymer-based | Polymers | Chang et al | Journal of Clinical Rehabilitative Tissue Engineering Research | 2008 |
| Haematology | Inorganic | Iron oxide NPs | Chen et al | Toxicology Letters | 2012 |
| Haematology | Polymer-based | Polymeric NPs | Chen et al | Journal of Nanoscience and Nanotechnology | 2015 |
| Haematology | Inorganic | Silver NPs | Chen et al | Chemical Research in Toxicology | 2015 |
| Haematology | Inorganic | Graphene oxide | Cheng et al | Biomacromolecules | 2012 |
| Haematology | Inorganic | Iron oxide NPs | Cheng et al | Biomaterials | 2005 |
| Haematology | Polymer-based | Polymeric NPs | Cherian et al | Biomedicine and Pharmacotherapy | 2015 |
| Haematology | Lipid-based | Lipid NPs | Chiang et al | Journal of Nanoparticle Research | 2011 |
| Haematology | Inorganic | Silica NPs | Cho et al | Particle and Fibre Toxicology | 2013 |
| Haematology | Inorganic | Titanium oxide NPs | Cho et al | Particle and Fibre Toxicology | 2013 |
| Haematology | Inorganic | Silver NPs | Choi et al | Toxicological Sciences | 2011 |
| Haematology | Inorganic | Graphene oxide NPs | Choimet et al | Colloids and Surfaces B: Biointerfaces | 2016 |
| Haematology | Inorganic | Graphene | Chowdhury et al | Scientific Reports | 2013 |
| Haematology | Polymer-based | Dendrimers | Ciolkowski et al | Nanomedicine: Nanotechnology, Biology, and Medicine | 2012 |
| Haematology | Polymer-based | Dendrimers | Ciolkowski et al | Biochimica et Biophysica Acta - Biomembranes | 2011 |
| Haematology | Inorganic | Cerium oxide NPs | Clark et al | Journal of Nanoparticle Research | 2011 |
| Haematology | Inorganic | Platinum NPs | Clark et al | Journal of Nanoparticle Research | 2011 |
| Haematology | Inorganic | Silver NPs | Cui et al | Materials Research Express | 2015 |
| Haematology | Inorganic | Hydroxyapatite NPs | Dai et al | Biological Trace Element Research | 2014 |
| Haematology | Polymer-based | Polymers | Das et al | Journal of Applied Polymer Science | 2008 |
| Haematology | Inorganic | Iron oxide | Das et al | Biomedical Materials | 2013 |
| Haematology | Polymer-based | Polymeric NPs | Davidson et al | ACS Biomaterials Science and Engineering | 2016 |
| Haematology | Polymer-based | Polymeric NPs | Deepagan et al | Nanomedicine | 2012 |
| Haematology | Inorganic | Silver NPs | Deka et al | Polymer Degradation and Stability | 2010 |
| Haematology | Inorganic | Gold NPs | Devendiran et al | Journal of Biomaterials and Tissue Engineering | 2014 |
| Haematology | Inorganic | Silver NPs | Devi et al | Journal of Physical Chemistry C | 2014 |
| Haematology | Inorganic | Carbon nanotubes | Diociaiuti et al | Environmental Research | 1999 |
| Haematology | Polymer-based | Dendrimers | Domański et al | Bioelectrochemistry | 2004 |
| Haematology | Inorganic | Titanium oxide | Dumitriu et al | Surface and Coatings Technology | 2015 |
| Haematology | Polymer-based | Polymeric NPs | Durán-Lobato et al | Journal of Nanoparticle Research | 2015 |
| Haematology | Lipid-based | Lipid NPs | Durán-Lobato et al | Journal of Nanoparticle Research | 2015 |
| Haematology | Inorganic | Apatite NPs | Dutta et al | Journal of Drug Targeting | 2007 |
| Haematology | Lipid-based | Lipid NPs | Eissa et al | PLoS ONE | 2015 |
| Haematology | Polymer-based | Dendrimers | El Kazzouli et al | Current Medicinal Chemistry | 2012 |
| Haematology | Inorganic | Iron oxide NPs | Ereath Beeran et al | Physical Chemistry Chemical Physics | 2015 |
| Haematology | Inorganic | Iron oxide NPs | Estevanato et al | International journal of nanomedicine | 2011 |
| Haematology | Polymer-based | Polymeric NPs | Farazuddin et al | International Journal of Nanomedicine | 2014 |
| Haematology | Polymer-based | Polymeric NPs | Farooq et al | Carbohydrate Polymers | 2017 |
| Haematology | Polymer-based | Polymeric NPs | Feuser et al | Journal of Nanoparticle Research | 2016 |
| Haematology | Polymer-based | Polymeric NPs | Figueiredo et al | Biomaterials | 2017 |
| Haematology | Inorganic | Iron oxide NPs | Figueiredo et al | Biomaterials | 2017 |
| Haematology | Polymer-based | Polymeric NPs | Forbes et al | European Journal of Pharmaceutics and Biopharmaceutics | 2013 |
| Haematology | Polymer-based | Polymeric NPs | Gaspar et al | Journal of Controlled Release | 2014 |
| Haematology | Polymer-based | Polymeric NPs | Gaudin et al | Nature Nanotechnology | 2014 |
| Haematology | Inorganic | Titanium oxide | Ghosh et al | Journal of Applied Toxicology | 2013 |
| Haematology | Polymer-based | Polymeric NPs | Gong et al | Journal of Nanoparticle Research | 2011 |
| Haematology | Inorganic | Gold NPs | Goshisht et al | ACS Sustainable Chemistry and Engineering | 2015 |
| Haematology | Polymer-based | Polymeric NPs | Gou et al | Pharmaceutical Research | 2009 |
| Haematology | Inorganic | Silica NPs | Greish et al | Nanotoxicology | 2012 |
| Haematology | Polymer-based | Dendrimers | Greish et al | Nanotoxicology | 2012 |
| Haematology | Inorganic | Silica NPs | Guo et al | ACS Applied Materials and Interfaces | 2015 |
| Haematology | Polymer-based | Polymeric NPs | Han et al | Langmuir | 2013 |
| Haematology | Inorganic | Iron oxide NPs | Herrmann et al | Nanomedicine | 2011 |
| Haematology | Inorganic | Gold NPs | Hess et al | Journal of Biomedical Materials Research-Part A | 2014 |
| Haematology | Inorganic | Platinum NPs | Hess et al | Journal of Biomedical Materials Research-Part A | 2014 |
| Haematology | Polymer-based | Polymeric NPs | Ho et al | Annals of Biomedical Engineering | 2016 |
| Haematology | Polymer-based | Polymeric NPs | Hu et al | Journal of Materials Chemistry | 2012 |
| Haematology | Polymer-based | Polymeric micelles | Huang et al | Journal of Materials Chemistry | 2011 |
| Haematology | Inorganic | Silver NPs | Huang et al | Scientific Reports | 2016 |
| Haematology | Polymer-based | Polymers | Imanieh et al | Systems and Synthetic Biology | 2013 |
| Haematology | Polymer-based | Dendrimers | Imran Ul-Haq et al | ACS Nano | 2013 |
| Haematology | Polymer-based | Polymeric NPs | Isaacman et al | Biomacromolecules | 2013 |
| Haematology | Polymer-based | Polymeric NPs | Jayalekshmi et al | Colloids and Surfaces B: Biointerfaces | 2013 |
| Haematology | Inorganic | Silica NPs | Jiang et al | Nanoscale Research Letters | 2016 |
| Haematology | Polymer-based | Polymeric NPs | Jin et al | Polymers for Advanced Technologies | 2013 |
| Haematology | Inorganic | Graphene oxide | Jin et al | Colloids and Surfaces B: Biointerfaces | 2013 |
| Haematology | Inorganic | Gadolinium NPs | Jin et al | New Journal of Chemistry | 2013 |
| Haematology | Lipid-based | Nanoemulsion | Jing et al | Nanomedicine: Nanotechnology, Biology, and Medicine | 2014 |
| Haematology | Lipid-based | Lipid NPs | Joshi et al | Nanomedicine | 2014 |
| Haematology | Inorganic | Silica NPs | Jovanovic et al | Biomacromolecules | 2006 |
| Haematology | Polymer-based | Polymers | Kapusetti et al | Journal of Materials Chemistry B | 2013 |
| Haematology | Polymer-based | Dendrimers | Kesharwani et al | Journal of Drug Delivery Science and Technology | 2015 |
| Haematology | Inorganic | Silica NPs | Kettiger et al | Biochimica et Biophysica Acta - Biomembranes | 2016 |
| Haematology | Inorganic | Silica NPs | Kettiger et al | Toxicology in Vitro | 2015 |
| Haematology | Inorganic | Gold NPs | Khullar et al | Journal of Physical Chemistry C | 2012 |
| Haematology | Inorganic | Silica NPs | Kim et al | Clinical Hemorheology and Microcirculation | 2016 |
| Haematology | Inorganic | Graphene NPs | Kim et al | Food and Chemical Toxicology | 2016 |
| Haematology | Polymer-based | Dendrimers | Klajnert et al | Bioelectrochemistry | 2004 |
| Haematology | Inorganic | Graphene oxide | Konwar et al | ACS Applied Materials and Interfaces | 2016 |
| Haematology | Polymer-based | Polymeric NPs | Kumar et al | Journal of Biomaterials and Tissue Engineering | 2014 |
| Haematology | Lipid-based | Lipid NPs | Kumar et al | Molecular Therapy - Nucleic Acids | 2014 |
| Haematology | Lipid-based | Liposomes | Kuznetsova et al | Journal of Controlled Release | 2012 |
| Haematology | Inorganic | Gold NPs | Lau et al | Nanotoxicology | 2012 |
| Haematology | Inorganic | Silver NPs | Li et al | Journal of Materials Chemistry B | 2015 |
| Haematology | Polymer-based | Polymers | Li et al | ACS Applied Materials and Interfaces | 2014 |
| Haematology | Polymer-based | Polymers | Li et al | ACS Applied Materials and Interfaces | 2015 |
| Haematology | Polymer-based | Polymeric NPs | Li et al | International Journal of Nanomedicine | 2012 |
| Haematology | Inorganic | Titanium oxide | Li et al | Journal of Materials Science: Materials in Medicine | 2014 |
| Haematology | Inorganic | Nanocrystals | Li et al | Scientific Reports | 2013 |
| Haematology | Inorganic | Vanadium dioxide NPs | Li et al | Nanoscale | 2016 |
| Haematology | Polymer-based | Polymeric NPs | Li et al | ACS Nano | 2014 |
| Haematology | Polymer-based | Polymeric NPs | Li et al | Biomaterials | 2014 |
| Haematology | Inorganic | Titanium oxide NPs | Li et al | Food and Chemical Toxicology | 2008 |
| Haematology | Polymer-based | Polymeric NPs | Liang et al | International Journal of Nanomedicine | 2014 |
| Haematology | Inorganic | Graphene oxide | Liao et al | ACS Applied Materials and Interfaces | 2011 |
| Haematology | Inorganic | Nanodiamonds | Lin et al | Journal of Biomedical Optics | 2012 |
| Haematology | Inorganic | Zinc oxide NPs | Lin et al | Journal of the American Chemical Society | 2010 |
| Haematology | Inorganic | Silica NPs | Lin et al | Chemistry of Materials | 2009 |
| Haematology | Inorganic | Mg Al Zn alloys | Liu et al | Journal of Biomedical Materials Research-Part A | 2015 |
| Haematology | Polymer-based | Polymeric NPs | Liu et al | Advanced Healthcare Materials | 2015 |
| Haematology | Inorganic | Nanocrystals | Liu et al | Journal of Pharmaceutical Sciences | 2010 |
| Haematology | Inorganic | Silica NPs | Liu et al | Chemical Communications | 2015 |
| Haematology | Inorganic | Halloysite nanotubes | Liu et al | Journal of Nanomaterials | 2015 |
| Haematology | Polymer-based | Polymers | Liu et al | Journal of Clinical Rehabilitative Tissue Engineering Research | 2008 |
| Haematology | Inorganic | Gold NPs | Liu et al | Journal of Materials Chemistry | 2012 |
| Haematology | Lipid-based | Liposomes | Lopes et al | European Journal of Pharmaceutics and Biopharmaceutics | 2012 |
| Haematology | Inorganic | Titanium oxide NPs | Lucky et al | ACS Nano | 2015 |
| Haematology | Lipid-based | Liposomes | Luo et al | Biomaterials Science | 2017 |
| Haematology | Inorganic | Graphene oxide | Ma et al | Journal of Colloid and Interface Science | 2017 |
| Haematology | Inorganic | Silica NPs | Ma et al | Journal of Colloid and Interface Science | 2016 |
| Haematology | Polymer-based | Polymers | Ma et al | Journal of Clinical Rehabilitative Tissue Engineering Research, 12 (14) | 2008 |
| Haematology | Polymer-based | Polymers | Ma et al | Journal of Clinical Rehabilitative Tissue Engineering Research, 12 (10) | 2008 |
| Haematology | Inorganic | Iron oxide NPs | Macías-Martínez et al | Journal of Applied Research and Technology | 2016 |
| Haematology | Inorganic | Gold NPs | Mahal et al | ACS Sustainable Chemistry and Engineering | 2013 |
| Haematology | Inorganic | Graphene oxide | Mahanta et al | Colloids and Surfaces B: Biointerfaces | 2015 |
| Haematology | Inorganic | Silica NPs | Malik et al | Journal of Controlled Release | 2000 |
| Haematology | Polymer-based | Dendrimers | Malik et al | Journal of Controlled Release | 2000 |
| Haematology | Inorganic | Silica NPs | Martinez et al | European Journal of Inorganic Chemistry | 2015 |
| Haematology | Polymer-based | Polymeric NPs | Maya et al | International Journal of Biological Macromolecules | 2012 |
| Haematology | Polymer-based | Polymeric NPs | Maya et al | Carbohydrate Polymers | 2013 |
| Haematology | Polymer-based | Polymeric NPs | Maya et al | Journal of Biomedical Nanotechnology | 2014 |
| Haematology | Polymer-based | Polymeric NPs | Mayer et al | Toxicology | 2009 |
| Haematology | Lipid-based | Nanoemulsion | Melariri et al | International Journal of Nanomedicine | 2015 |
| Haematology | Inorganic | Carbon nanotubes | Meng et al | PLoS ONE | 2012 |
| Haematology | Inorganic | Iron oxide | Milowska et al | ACS Applied Materials and Interfaces | 2015 |
| Haematology | Lipid-based | Liposomes | Morizawa et al | Biomaterials, Artificial Cells and Immobilization Biotechnology | 1992 |
| Haematology | Polymer-based | Polymeric NPs | Narayanan et al | Acta Biomaterialia | 2014 |
| Haematology | Inorganic | Silica NPs | Nemmar et al | Cellular Physiology and Biochemistry | 2014 |
| Haematology | Polymer-based | Micelles | Oberoi et al | International Journal of Nanomedicine | 2012 |
| Haematology | Polymer-based | Polymeric NPs | Paillard et al | Pharmaceutical Research | 2010 |
| Haematology | Inorganic | Quantum dots | Painuly et al | Journal of Biomaterials Applications | 2014 |
| Haematology | Inorganic | Hydroxyapatite NPs | Palanivelu et al | Spectrochimica Acta - Part A: Molecular and Biomolecular Spectroscopy | 2014 |
| Haematology | Inorganic | Iron oxide NPs | Passemard et al | Bioorganic and Medicinal Chemistry Letters | 2013 |
| Haematology | Lipid-based | Liposomes | Patel et al | Nanomedicine | 2016 |
| Haematology | Lipid-based | Liposomes | Patel et al | Journal of Nanoparticle Research | 2014 |
| Haematology | Polymer-based | Polymers | Paul et al | International Journal of ChemTech Research | 2015 |
| Haematology | Inorganic | Silica NPs | Paula et al | Journal of the Brazilian Chemical Society | 2012 |
| Haematology | Inorganic | Silver NPs | Pavan et al | Particle and Fibre Toxicology | 2014 |
| Haematology | Inorganic | Silver NPs | Pavan et al | Chemical Research in Toxicology | 2013 |
| Haematology | Polymer-based | Polymeric NPs | Peng et al | Biomaterials Science | 2016 |
| Haematology | Polymer-based | Nanogels | Pereira et al | Toxicology in Vitro | 2015 |
| Haematology | Lipid-based | Liposomes | Perumal et al | Cancer Nanotechnology | 2011 |
| Haematology | Inorganic | Graphene | Pinto et al | Carbon | 2016 |
| Haematology | Inorganic | Iron oxide NPs | Prabu et al | Nanoscale | 2015 |
| Haematology | Inorganic | Carbon nanotubes | Prodana et al | Ceramics International | 2015 |
| Haematology | Inorganic | Silica NPs | Pu et al | ChemPlusChem | 2013 |
| Haematology | Polymer-based | Polymers | Ramana Ramya et al | Ceramics International | 2016 |
| Haematology | Inorganic | Hydroxyapatite NPs | Ramya et al | Ceramics International | 2014 |
| Haematology | Inorganic | Gold NPs | Rani et al | RSC Advances | 2016 |
| Haematology | Inorganic | Gold NPs | Razzaboni et al | Environmental Health Perspectives | 1990 |
| Haematology | Polymer-based | Nanogels | Rejinold et al | Colloids and Surfaces B: Biointerfaces | 2014 |
| Haematology | Polymer-based | Polymeric NPs | Ribeiro et al | International Journal of Nanomedicine | 2014 |
| Haematology | Inorganic | Quantum dots | Rocha et al | Marine Environmental Research | 2014 |
| Haematology | Polymer-based | Polymeric NPs | Sahiner et al | Polymer Degradation and Stability | 2016 |
| Haematology | Lipid-based | Liposomes | Salzano et al | Journal of Biomedical Nanotechnology | 2016 |
| Haematology | Inorganic | Gold NPs | Sasidharan et al | ACS Biomaterials Science and Engineering | 2016 |
| Haematology | Inorganic | Graphene | Sasidharan et al | Small | 2012 |
| Haematology | Polymer-based | Dendrimers | Sathishkumar et al | RSC Advances | 2015 |
| Haematology | Inorganic | Hydroxyapatite NPs | Selvakumar et al | Journal of Biomedical Nanotechnology | 2015 |
| Haematology | Inorganic | Hydroxyapatite NPs | Selvakumar et al | Crystal Growth and Design | 2017 |
| Haematology | Inorganic | Silver NPs | Semenova et al | Journal of Materials Chemistry | 2012 |
| Haematology | Polymer-based | Polymeric NPs | Shah et al | International Journal of Pharmaceutics | 2000 |
| Haematology | Inorganic | Silica NPs | Shahbazi et al | Biomaterials | 2013 |
| Haematology | Inorganic | Iron oxide NPs | Shen et al | Nanotechnology | 2012 |
| Haematology | Inorganic | Carbon nanotubes | Shi et al | RSC Advances | 2016 |
| Haematology | Inorganic | Gold NPs | Shi et al | Acta Biomaterialia | 2012 |
| Haematology | Inorganic | Silica NPs | Shi et al | Acta Biomaterialia | 2012 |
| Haematology | Polymer-based | Polymers | Singh et al | Journal of Materials Chemistry | 2012 |
| Haematology | Inorganic | Graphene | Singh et al | ACS Nano | 2012 |
| Haematology | Inorganic | Gold NPs | Singh et al | ACS Sustainable Chemistry and Engineering | 2013 |
| Haematology | Inorganic | Nanodiamonds | Skoog et al | Journal of Biomedical Materials Research-Part A | 2017 |
| Haematology | Inorganic | Silica NPs | Slowing et al | Small | 2009 |
| Haematology | Inorganic | Bismuth Ferrite NPs | Staedler et al | Nanomedicine: Nanotechnology, Biology, and Medicine | 2014 |
| Haematology | Inorganic | Silver NPs | Su et al | Colloids and Surfaces B: Biointerfaces | 2017 |
| Haematology | Polymer-based | Dendrimers | Swami et al | Journal of Nanoparticle Research | 2015 |
| Haematology | Inorganic | Iron oxide NPs | Szczeszak et al | Journal of Nanoparticle Research | 2015 |
| Haematology | Lipid-based | Liposomes | Tagami et al | Journal of Controlled Release | 2011 |
| Haematology | Inorganic | Iron oxide NPs | Tan et al | Materials Science and Engineering C | 2016 |
| Haematology | Inorganic | Hydroxyapatite | Tang et al | Journal of Clinical Rehabilitative Tissue Engineering Research | 2010 |
| Haematology | Inorganic | Silica NPs | Tavano et al | Nanomedicine | 2010 |
| Haematology | Polymer-based | Dendrimers | Tekade et al | Pharmaceutical Research | 2015 |
| Haematology | Inorganic | Silica NPs | Teng et al | Small | 2016 |
| Haematology | Inorganic | Silica NPs | Tenzer et al | Nature Nanotechnology | 2013 |
| Haematology | Inorganic | Graphene oxide | Thampi et al | Journal of Applied Polymer Science | 2015 |
| Haematology | Polymer-based | Polymeric NPs | Tong et al | Journal of Materials Chemistry B | 2013 |
| Haematology | Inorganic | Fullerene NPs | Trpkovic et al | Nanotechnology | 2010 |
| Haematology | Inorganic | Titanium oxide NPs | Ungureanu et al | Bioelectrochemistry | 2016 |
| Haematology | Polymer-based | Polymeric NPs | Urbán et al | Journal of Controlled Release | 2014 |
| Haematology | Inorganic | Iron oxide NPs | Valois et al | Biomaterials | 2010 |
| Haematology | Polymer-based | Polymeric NPs | Van De Ven et al | Journal of Controlled Release | 2012 |
| Haematology | Polymer-based | Polymers | Venault et al | Journal of Membrane Science | 2014 |
| Haematology | Polymer-based | Nanogels | Vijayan et al | Colloids and Surfaces B: Biointerfaces | 2015 |
| Haematology | Polymer-based | Nanogels | Vijayan et al | Colloids and Surfaces B: Biointerfaces | 2016 |
| Haematology | Inorganic | Aluminum oxide NPs | Vinardell et al | Journal of Nanoparticle Research | 2015 |
| Haematology | Polymer-based | Polymeric NPs | Vora et al | Journal of Nanoparticle Research | 2014 |
| Haematology | Inorganic | Silica NPs | Wang et al | Dalton Transactions | 2013 |
| Haematology | Inorganic | Zirconia NPs | Wang et al | Scientific Reports | 2016 |
| Haematology | Inorganic | Hydroxyapatite NPs | Wang et al | International journal of nanomedicine | 2011 |
| Haematology | Polymer-based | Polymeric NPs | Weiss et al | Journal of Applied Biomaterials and Functional Materials | 2012 |
| Haematology | Inorganic | Cobalt ferrite NPs | Wu et al | Acta Biomaterialia | 2011 |
| Haematology | Polymer-based | Nanogels | Wu et al | Biomacromolecules | 2013 |
| Haematology | Inorganic | Iron oxide NPs | Wu et al | Chemistry - A European Journal | 2007 |
| Haematology | Polymer-based | Polymeric NPs | Xie et al | International Journal of Pharmaceutics | 2015 |
| Haematology | Polymer-based | Polymers | Xiong et al | International Journal of Nanomedicine | 2014 |
| Haematology | Inorganic | Gold NPs | Xu et al | Molecular Pharmaceutics | 2016 |
| Haematology | Inorganic | Silver NPs | Xu et al | Molecular Pharmaceutics | 2016 |
| Haematology | Inorganic | Nanocrystals | Xu et al | ACS Applied Materials and Interfaces | 2012 |
| Haematology | Polymer-based | Polymeric NPs | Yadav et al | Journal of Materials Chemistry B | 2014 |
| Haematology | Inorganic | Iron oxide NPs | Yallapu et al | Biomaterials | 2015 |
| Haematology | Polymer-based | Polymers | Yan et al | Materials Technology | 2016 |
| Haematology | Inorganic | Magnetosomes | Yan et al | Materials Science and Engineering C | 2012 |
| Haematology | Polymer-based | Polymeric NPs | Yang et al | International Journal of Nanomedicine | 2012 |
| Haematology | Polymer-based | Micelles | Yang et al | Journal of Pharmaceutical Sciences | 2014 |
| Haematology | Inorganic | TiO2 nanotubes | Yang et al | Colloids and Surfaces B: Biointerfaces | 2010 |
| Haematology | Polymer-based | Dendrimers | Yellepeddi et al | Expert Opinion on Drug Delivery | 2009 |
| Haematology | Inorganic | Silica NPs | Yildirim et al | Advanced Healthcare Materials | 2016 |
| Haematology | Inorganic | Silica NPs | Yildirim et al | Journal of Materials Chemistry B | 2013 |
| Haematology | Inorganic | Silica NPs | Yu et al | ACS Nano | 2011 |
| Haematology | Inorganic | Silver NPs | Zare-Zardini et al | Journal of Biomedical Materials Research-Part A | 2015 |
| Haematology | Inorganic | Carbon nanotubes | Zare-Zardini et al | Journal of Biomedical Materials Research-Part A | 2015 |
| Haematology | Inorganic | Nanocrystals | Zhang et al | CrystEngComm | 2012 |
| Haematology | Inorganic | Hydroxyapatite NPs | Zhang et al | Journal of Clinical Rehabilitative Tissue Engineering Research | 2008 |
| Haematology | Inorganic | Carbon dots | Zhang et al | Journal of Nanoparticle Research | 2013 |
| Haematology | Inorganic | Carbon NPs | Zhang et al | Nanoscale | 2015 |
| Haematology | Inorganic | Zinc oxide NPs | Zhao et al | CrystEngComm | 2012 |
| Haematology | Inorganic | Gold NPs | Zhao et al | ACS Nano | 2011 |
| Haematology | Inorganic | Silver NPs | Zhou et al | Dalton Transactions | 2014 |
| Haematology | Polymer-based | Polymers | Zhou et al | Journal of Macromolecular Science, Part A: Pure and Applied Chemistry | 2014 |
| Coagulation | Inorganic | Silica NPs | Abraham et al | Biomacromolecules | 2011 |
| Coagulation | Polymer-based | Dendrimers | Alavidjeh et al | Journal of Materials Science: Materials in Medicine | 2010 |
| Coagulation | Inorganic | Iron oxide NPs | Angelova et al | Colloids and Surfaces A: Physicochemical and Engineering Aspects | 2017 |
| Coagulation | Polymer-based | Polymeric NPs | Anitha et al | Journal of Biomedical Nanotechnology | 2012 |
| Coagulation | Polymer-based | Nanogels | Arunraj et al | Colloids and Surfaces B: Biointerfaces | 2014 |
| Coagulation | Polymer-based | Nanogels | Arunraj et al | International Journal of Biological Macromolecules | 2013 |
| Coagulation | Polymer-based | Polymeric NPs | Brambilla et al | ACS Nano | 2012 |
| Coagulation | Inorganic | Carbon nanotubes | Burke et al | Biomaterials | 2011 |
| Coagulation | Polymer-based | Polymeric NPs | Chen et al | Journal of Nanoscience and Nanotechnology | 2015 |
| Coagulation | Inorganic | Silica NPs | Chen et al | ACS Nano | 2010 |
| Coagulation | Inorganic | Iron oxide NPs | Crespo et al | Pharmaceutical Research | 2017 |
| Coagulation | Polymer-based | Nanogels | Easo et al | Colloids and Surfaces B: Biointerfaces | 2015 |
| Coagulation | Lipid-based | Liposomes | Gaffney et al | Nanomedicine: Nanotechnology, Biology, and Medicine | 2015 |
| Coagulation | Inorganic | Silica NPs | Gryshchuk et al | Biochemistry Research International | 2016 |
| Coagulation | Inorganic | Silver NPs | Guildford et al | Journal of the Royal Society Interface | 2009 |
| Coagulation | Inorganic | Titanium oxide NPs | Guildford et al | Journal of the Royal Society Interface | 2009 |
| Coagulation | Inorganic | Iron oxide NPs | Guildford et al | Journal of the Royal Society Interface | 2009 |
| Coagulation | Polymer-based | Polymeric NPs | Han et al | Langmuir | 2013 |
| Coagulation | Polymer-based | Polymeric micelles | Herrmann et al | Nanomedicine | 2011 |
| Coagulation | Inorganic | Hydroxide NPs | Hu et al | Journal of Materials Chemistry | 2012 |
| Coagulation | Inorganic | Silica NPs | Huang et al | Journal of Materials Chemistry | 2011 |
| Coagulation | Inorganic | Silica NPs | Imran Ul-Haq et al | ACS Nano | 2013 |
| Coagulation | Inorganic | Silica NPs | Jovanovic et al | Biomacromolecules | 2006 |
| Coagulation | Inorganic | Silver NPs | Jun et al | Nanotoxicology | 2011 |
| Coagulation | Polymer-based | Polymers | Kainthan et al | Biomaterials | 2007 |
| Coagulation | Lipid-based | Liposomes | Kuznetsova et al | Journal of Controlled Release | 2012 |
| Coagulation | Inorganic | Iron oxide NPs | Kuznetsova et al | Biochemistry (Moscow) | 2014 |
| Coagulation | Inorganic | Silver NPs | Laloy et al | Nanomaterials and Nanotechnology | 2014 |
| Coagulation | Polymer-based | Hydrogels | Li et al | ACS Applied Materials and Interfaces | 2015 |
| Coagulation | Lipid-based | Liposomes | Li et al | Chinese Pharmaceutical Journal | 2015 |
| Coagulation | Polymer-based | Polymeric NPs | Liu et al | Advanced Healthcare Materials | 2015 |
| Coagulation | Inorganic | Silver NPs | Major et al | IOP Conference Series: Materials Science and Engineering | 2016 |
| Coagulation | Inorganic | Silver NPs | Martínez-Gutierrez et al | Nanomedicine: Nanotechnology, Biology, and Medicine | 2012 |
| Coagulation | Inorganic | Carbon nanotubes | Meng et al | PLoS ONE | 2012 |
| Coagulation | Inorganic | Silica NPs | Minet et al | Journal of Nanoparticle Research | 2015 |
| Coagulation | Inorganic | Silver NPs | Minet et al | Journal of Nanoparticle Research | 2015 |
| Coagulation | Inorganic | Silica NPs | Nemmar et al | International Journal of Nanomedicine | 2014 |
| Coagulation | Inorganic | Iron oxide NPs | Nemmar et al | Part Fibre Toxicol | 2016 |
| Coagulation | Lipid-based | Liposomes | Pinnapireddy et al | Colloids and Surfaces B: Biointerfaces | 2017 |
| Coagulation | Inorganic | Gold NPs | Sasidharan et al | ACS Biomaterials Science and Engineering | 2016 |
| Coagulation | Polymer-based | Dendrimers | Shenoi et al | Journal of the American Chemical Society | 2012 |
| Coagulation | Inorganic | Iron oxide NPs | Simberg et al | Journal of Controlled Release | 2009 |
| Coagulation | Inorganic | Silver NPs | Steuer et al | Journal of Biomedical Materials Research - Part B Applied Biomaterials | 2014 |
| Coagulation | Polymer-based | Polymeric NPs | Sun et al | Analytica Chimica Acta | 2013 |
| Coagulation | Inorganic | Silica NPs | Tavano et al | Nanomedicine | 2010 |
| Coagulation | Inorganic | Carbon nanotubes | Vakhrusheva et al | Toxicology Letters | 2013 |
| Coagulation | Inorganic | Iron oxide NPs | Wuang et al | Advanced Functional Materials | 2006 |
| Coagulation | Polymer-based | Nanogel | Xia et al | Polymer Chemistry | 2014 |
| Coagulation | Inorganic | Silica NPs | Yildirim et al | Journal of Materials Chemistry B | 2013 |
| Coagulation | Polymer-based | Polymeric NPs | Zarrabi et al | Journal of Materials Science: Materials in Medicine | 2014 |
| Platelets | Polymer-based | Polymeric NPs | Anselmo et al | ACS Nano | 2014 |
| Platelets | Inorganic | Zinc oxide NPs | Aula et al | Materials Research Express | 2014 |
| Platelets | Polymer-based | Polymeric NPs | Brambilla et al | ACS Nano | 2012 |
| Platelets | Inorganic | Carbon nanotubes | Burke et al | Biomaterials | 2011 |
| Platelets | Inorganic | Gold NPs | Chanda et al | Nanomedicine: Nanotechnology, Biology, and Medicine | 2010 |
| Platelets | Lipid-based | Liposomes | Cheng et al | Advanced Healthcare Materials | 2016 |
| Platelets | Inorganic | Graphene | Chowdhury et al | Scientific Reports | 2013 |
| Platelets | Inorganic | Boron nitride nanotubes | Ciofani et al | International Journal of Nanomedicine | 2012 |
| Platelets | Polymer-based | Polymers | Colvin et al | Modern Plastics Worldwide | 2007 |
| Platelets | Inorganic | Iron oxide NPs | Comănescu et al | Romanian Journal of Morphology and Embryology | 2015 |
| Platelets | Polymer-based | Polymers | Coombes et al | Biomaterials | 2001 |
| Platelets | Inorganic | Silica NPs | Corbalan et al | International Journal of Nanomedicine | 2012 |
| Platelets | Polymer-based | Dendrimers | Dobrovolskaia et al | Molecular Pharmaceutics | 2012 |
| Platelets | Inorganic | Quantum dots | Dunpall et al | IUBMB Life | 2012 |
| Platelets | Polymer-based | Dendrimers | Enciso et al | Molecules | 2016 |
| Platelets | Inorganic | Carbon nanotubes | Fent et al | Toxicology in Vitro | 2015 |
| Platelets | Inorganic | Carbon nanotubes | Gaffney et al | Nanomedicine: Nanotechnology, Biology, and Medicine | 2015 |
| Platelets | Inorganic | Silica NPs | Gryshchuk et al | Biochemistry Research International | 2016 |
| Platelets | Inorganic | Silica NPs | Guidetti et al | Nanomedicine: Nanotechnology, Biology, and Medicine | 2012 |
| Platelets | Inorganic | Carbon nanotubes | Guidetti et al | Nanomedicine: Nanotechnology, Biology, and Medicine | 2012 |
| Platelets | Inorganic | Silver NPs | Guildford et al | Journal of the Royal Society Interface | 2009 |
| Platelets | Inorganic | Titanium oxide NPs | Guildford et al | Journal of the Royal Society Interface | 2009 |
| Platelets | Polymer-based | Polymeric NPs | Han et al | Langmuir | 2013 |
| Platelets | Polymer-based | Hydrogels | He et al | Journal of Materials Chemistry B | 2015 |
| Platelets | Inorganic | Iron oxide NPs | Herrmann et al | Nanomedicine | 2011 |
| Platelets | Inorganic | Gold NPs | Hess et al | Journal of Biomedical Materials Research-Part A | 2014 |
| Platelets | Inorganic | Platinum NPs | Hess et al | Journal of Biomedical Materials Research-Part A | 2014 |
| Platelets | Polymer-based | Polymers | Honarbakhsh et al | Journal of Materials Science | 2011 |
| Platelets | Inorganic | Hydroxyde NPs | Hu et al | Journal of Materials Chemistry | 2012 |
| Platelets | Polymer-based | Dendrimers | Imran Ul-Haq et al | ACS Nano | 2013 |
| Platelets | Inorganic | Graphene | Jin et al | Colloids and Surfaces B: Biointerfaces | 2013 |
| Platelets | Polymer-based | Dendrimers | Jones et al | ACS Nano | 2012 |
| Platelets | Polymer-based | Dendrimers | Jones et al | Molecular Pharmaceutics | 2012 |
| Platelets | Lipid-based | Liposomes | Juliano et al | Experimental Cell Research | 1983 |
| Platelets | Polymer-based | Polymers | Kainthan et al | Biomaterials | 2007 |
| Platelets | Inorganic | Titanium oxide NPs | Karagkiozaki et al | International Journal of Nanomedicine | 2012 |
| Platelets | Inorganic | Carbon nanotubes | Karagkiozaki et al | International Journal of Nanomedicine | 2012 |
| Platelets | Inorganic | Titanium oxide NPs | Karagkiozaki et al | Nanomedicine: Nanotechnology, Biology, and Medicine | 2009 |
| Platelets | Lipid-based | Liposomes | Khandelwal et al | RSC Advances | 2015 |
| Platelets | Inorganic | Gold NPs | Kumar et al | Process Biochemistry | 2011 |
| Platelets | Inorganic | Nanodiamonds | Kumari et al | Nanomedicine | 2014 |
| Platelets | Inorganic | Silicon nanowires | Lagonegro et al | Materials Science and Engineering C | 2017 |
| Platelets | Polymer-based | Polymers | Lai et al | Biomaterials | 2010 |
| Platelets | Lipid-based | Liposomes | Lapenda et al | Journal of Biomedical Nanotechnology | 2013 |
| Platelets | Inorganic | Diamond-like carbon | Leng et al | Surface Science | 2003 |
| Platelets | Inorganic | Carbon nanotubes | Li et al | Russian Journal of Inorganic Chemistry | 2011 |
| Platelets | Polymer-based | Polymers | Lim et al | Applied Surface Science | 2014 |
| Platelets | Polymer-based | Polymers | Liu et al | Journal of Clinical Rehabilitative Tissue Engineering Research | 2008 |
| Platelets | Polymer-based | Polymeric NPs | Mayer et al | Toxicology | 2009 |
| Platelets | Lipid-based | Micelles | Mei et al | Biomaterials | 2015 |
| Platelets | Inorganic | Carbon nanotubes | Meng et al | PLoS ONE | 2012 |
| Platelets | Inorganic | Hydroxyapatite NPs | Miller et al | Nanomedicine | 2009 |
| Platelets | Polymer-based | Polymers | Minelli et al | Journal of Nanobiotechnology | 2008 |
| Platelets | Inorganic | Diamond-like carbon | Nandakumar et al | ACS Applied Materials and Interfaces | 2016 |
| Platelets | Inorganic | Titanium oxide NPs | Nandakumar et al | ACS Applied Materials and Interfaces | 2016 |
| Platelets | Inorganic | Silica NPs | Nemmar et al | International Journal of Nanomedicine | 2014 |
| Platelets | Inorganic | Iron oxide NPs | Nemmar et al | Particle and Fibre Toxicology | 2016 |
| Platelets | Polymer-based | Polymers | Nie et al | Biomacromolecules | 2015 |
| Platelets | Inorganic | Diamond-like carbon | Okpalugo et al | Diamond and Related Materials | 2004 |
| Platelets | Polymer-based | Polymeric NPs | Oommen et al | ACS Applied Materials and Interfaces | 2016 |
| Platelets | Polymer-based | Polymeric NPs | Peng et al | Biomaterials Science | 2016 |
| Platelets | Inorganic | Carbon NPs | Radomski et al | British Journal of Pharmacology | 2005 |
| Platelets | Inorganic | Carbon nanotubes | Radomski et al | British Journal of Pharmacology | 2005 |
| Platelets | Inorganic | Silver NPs | Ragaseema et al | Biomaterials | 2012 |
| Platelets | Inorganic | Silica NPs | Sadaf et al | Journal of Nanoscience and Nanotechnology | 2012 |
| Platelets | Inorganic | Quantum dots | Sadaf et al | Journal of Nanoscience and Nanotechnology | 2012 |
| Platelets | Polymer-based | Hydrogels | Saini et al | Acta Biomaterialia | 2016 |
| Platelets | Inorganic | Gold NPs | Santos-Martinez et al | Journal of Biomedical Nanotechnology | 2014 |
| Platelets | Inorganic | Gold NPs | Sasidharan et al | ACS Biomaterials Science and Engineering | 2016 |
| Platelets | Inorganic | Graphene | Sasidharan et al | Small | 2012 |
| Platelets | Polymer-based | Polymeric NPs | Sasidharan et al | ACS Applied Materials and Interfaces | 2016 |
| Platelets | Polymer-based | Dendrimers | Shenoi et al | Journal of the American Chemical Society | 2012 |
| Platelets | Inorganic | Silver NPs | Shrivastava et al | ACS Nano | 2009 |
| Platelets | Inorganic | Silver NPs | Shrivastava et al | Colloids and Surfaces B: Biointerfaces | 2011 |
| Platelets | Polymer-based | Polymers | Shukla et al | Journal of Macromolecular Science, Part A: Pure and Applied Chemistry | 2010 |
| Platelets | Polymer-based | Polymeric NPs | Singh et al | Journal of Materials Chemistry | 2012 |
| Platelets | Inorganic | Graphene oxide | Singh et al | ACS Nano | 2012 |
| Platelets | Inorganic | Graphene oxide | Singh et al | ACS Nano | 2011 |
| Platelets | Inorganic | Nanodiamonds | Skoog et al | Journal of Biomedical Materials Research-Part A | 2017 |
| Platelets | Inorganic | Titanium nanotubes | Smith et al | Journal of Biomedical Nanotechnology | 2012 |
| Platelets | Inorganic | Silver NPs | Sriram et al | International Journal of Nanomedicine | 2010 |
| Platelets | Inorganic | Iron oxide NPs | Stamopoulos et al | Current Nanoscience | 2009 |
| Platelets | Inorganic | Graphene oxide | Stevens et al | ACS Applied Materials and Interfaces | 2009 |
| Platelets | Polymer-based | Polymeric NPs | Sun et al | Analytica Chimica Acta | 2013 |
| Platelets | Inorganic | Carbon nanotubes | Sun et al | Small | 2005 |
| Platelets | Inorganic | Carbon nanotubes | Takahashi et al | Journal of Biorheology | 2009 |
| Platelets | Inorganic | Silica NPs | Tavano et al | Nanomedicine | 2010 |
| Platelets | Polymer-based | Polymeric NPs | Tong et al | Journal of Materials Chemistry B | 2013 |
| Platelets | Inorganic | Carbon nanotubes | Vakhrusheva et al | Toxicology Letters | 2013 |
| Platelets | Lipid-based | Lipid NPs | Wauthoz et al | Nanomedicine: Nanotechnology, Biology, and Medicine | 2015 |
| Platelets | Inorganic | Silicon dioxide | Weisenberg et al | Journal of Biomedical Materials Research | 2002 |
| Platelets | Polymer-based | Polymers | Weisenberg et al | Journal of Biomedical Materials Research | 2002 |
| Platelets | Inorganic | Titanium nanotubes | Yang et al | Colloids and Surfaces B: Biointerfaces | 2010 |
| Platelets | Polymer-based | Polymeric NPs | Ye et al | Applied Surface Science | 2015 |
| Platelets | Polymer-based | Polymers | Yue et al | Journal of biomedical engineering | 2008 |
| Platelets | Lipid-based | Liposomes | Zhang et al | Theranostics | 2017 |
| Complement | Polymer-based | Dendrimers | Åkesson et al | RSC Advances | 2012 |
| Complement | Inorganic | Iron oxide NPs | Allard-Vannier et al | European Journal of Pharmaceutics and Biopharmaceutics | 2012 |
| Complement | Polymer-based | Polymeric NPs | Allémann et al | Journal of Biomedical Materials Research | 1997 |
| Complement | Inorganic | Carbon nanotubes | Andersen et al | Nanomedicine: Nanotechnology, Biology, and Medicine | 2013 |
| Complement | Inorganic | Iron oxide NPs | Aqil et al | European Polymer Journal | 2008 |
| Complement | Inorganic | Iron oxide NPs | Bellido et al | Advanced Healthcare Materials | 2015 |
| Complement | Polymer-based | Polymeric NPs | Bertholon et al | Pharmaceutical Research | 2006 |
| Complement | Polymer-based | Polymeric NPs | Brambilla et al | ACS Nano | 2012 |
| Complement | Inorganic | Carbon nanotubes | Bussy et al | Advanced Drug Delivery Reviews | 2013 |
| Complement | Polymer-based | Polymeric NPs | Cenni et al | Biomaterials | 2008 |
| Complement | Inorganic | Gold NPs | Chanda et al | Nanomedicine: Nanotechnology, Biology, and Medicine | 2010 |
| Complement | Polymer-based | Polymeric NPs | Chauvierre et al | Biomaterials | 2010 |
| Complement | Polymer-based | Polymeric NPs | Chen et al | Journal of Nanoscience and Nanotechnology | 2015 |
| Complement | Inorganic | Graphene | Chowdhury et al | Scientific Reports | 2013 |
| Complement | Polymer-based | Polymeric NPs | Dash et al | Biomaterials | 2010 |
| Complement | Lipid-based | Liposomes | Dézsi et al | Journal of Controlled Release | 2014 |
| Complement | Polymer-based | Micelles | Donev et al | World Journal of Biological Psychiatry | 2011 |
| Complement | Polymer-based | Polymeric NPs | Donev et al | World Journal of Biological Psychiatry | 2011 |
| Complement | Polymer-based | Dendrimers | Donev et al | World Journal of Biological Psychiatry | 2011 |
| Complement | Inorganic | Carbon nanotubes | Dvash et al | Journal of Controlled Release | 2013 |
| Complement | Polymer-based | Polymeric NPs | Eidi et al | International Journal of Pharmaceutics | 2010 |
| Complement | Polymer-based | Polymeric NPs | Engberg et al | Journal of Biomedical Materials Research-Part A | 2011 |
| Complement | Inorganic | Titanium oxide | Fischer et al | Journal of Biomedical Materials Research-Part A | 2007 |
| Complement | Inorganic | Zirconia | Fischer et al | Journal of Biomedical Materials Research-Part A | 2007 |
| Complement | Inorganic | Alumina | Fischer et al | Journal of Biomedical Materials Research-Part A | 2007 |
| Complement | Polymer-based | Polymers | Fischer et al | Journal of Biomedical Materials Research-Part A | 2007 |
| Complement | Polymer-based | Polymeric NPs | Gaucher et al | Biomacromolecules | 2009 |
| Complement | Polymer-based | Polymeric NPs | Gaudin et al | Nature Nanotechnology | 2014 |
| Complement | Lipid-based | Lipid NPs | Hak et al | Pharmaceutical Research | 2015 |
| Complement | Polymer-based | Polymeric NPs | Hamad et al | ACS Nano | 2010 |
| Complement | Inorganic | Carbon nanotubes | Hamad et al | Molecular Immunology | 2008 |
| Complement | Inorganic | Carbon nanotubes | Hamad et al | Molecular Immunology | 2008 |
| Complement | Polymer-based | Polymeric NPs | Han et al | Langmuir | 2013 |
| Complement | Inorganic | Iron oxide NPs | Herrmann et al | Nanomedicine | 2011 |
| Complement | Lipid-based | Lipid NPs | Hirsjärvi et al | Acta Biomaterialia | 2013 |
| Complement | Lipid-based | Lipid NPs | Hirsjärvi et al | Nanomedicine: Nanotechnology, Biology, and Medicine | 2013 |
| Complement | Lipid-based | Nanoemulsion | Hirsjärvi et al | Nanomedicine: Nanotechnology, Biology, and Medicine | 2013 |
| Complement | Polymer-based | Polymeric NPs | Hu et al | Nature | 2015 |
| Complement | Inorganic | Silver NPs | Huang et al | Scientific Reports | 2016 |
| Complement | Inorganic | Gold NPs | Hulander et al | International Journal of Nanomedicine | 2011 |
| Complement | Lipid-based | Lipid NPs | Huynh et al | Pharmaceutical Research | 2011 |
| Complement | Polymer-based | Dendrimers | Imran Ul-Haq et al | ACS Nano | 2013 |
| Complement | Polymer-based | Polymeric NPs | Isaacman et al | Biomacromolecules | 2013 |
| Complement | Polymer-based | Polymeric NPs | Kainthan et al | Biomaterials | 2006 |
| Complement | Polymer-based | Polymeric NPs | Kainthan et al | Biomaterials | 2007 |
| Complement | Lipid-based | Lipid NPs | Kheir et al | Advanced Healthcare Materials | 2013 |
| Complement | Inorganic | Silver NPs | Kumar et al | BioNanoScience | 2012 |
| Complement | Inorganic | Silver NPs | Kumar et al | Process Biochemistry | 2011 |
| Complement | Lipid-based | Liposomes | Kuznetsova et al | Journal of Controlled Release | 2012 |
| Complement | Lipid-based | Liposomes | Kuznetsova et al | Biochemistry (Moscow) | 2014 |
| Complement | Polymer-based | Polymeric NPs | Labarre et al | Biomaterials | 2005 |
| Complement | Polymer-based | Polymeric NPs | Layre et al | Journal of Controlled Release | 2006 |
| Complement | Polymer-based | Polymers | Leszczak et al | ACS Applied Materials and Interfaces | 2014 |
| Complement | Inorganic | Carbon nanotubes | Ling et al | ACS Nano | 2011 |
| Complement | Inorganic | Silver NPs | Long et al | Nanotoxicology | 2016 |
| Complement | Polymer-based | Polymers | Ma et al | Journal of Clinical Rehabilitative Tissue Engineering Research | 2008 |
| Complement | Polymer-based | Polymeric NPs | Martínez-Barbosa et al | Bioconjugate Chemistry | 2009 |
| Complement | Polymer-based | Polymeric NPs | Mayer et al | Toxicology | 2009 |
| Complement | Lipid-based | Liposomes | Mészáros et al | Nanomedicine: Nanotechnology, Biology, and Medicine | 2016 |
| Complement | Lipid-based | Micelles | Mészáros et al | Nanomedicine: Nanotechnology, Biology, and Medicine | 2016 |
| Complement | Polymer-based | Polymeric NPs | Misra et al | Molecular Pharmaceutics | 2014 |
| Complement | Polymer-based | Polymeric NPs | Mizrahy et al | Journal of Controlled Release | 2011 |
| Complement | Lipid-based | Liposomes | Moghimi et al | Journal of Controlled Release | 2010 |
| Complement | Lipid-based | Micelles | Moghimi et al | Journal of Controlled Release | 2010 |
| Complement | Inorganic | Carbon nanotubes | Moghimi et al | Journal of Controlled Release | 2010 |
| Complement | Polymer-based | Polymeric NPs | Molino et al | Biomacromolecules | 2012 |
| Complement | Polymer-based | Polymeric NPs | Mosqueira et al | Biomaterials | 2001 |
| Complement | Lipid-based | Lipid NPs | Mosqueira et al | Biomaterials | 2001 |
| Complement | Inorganic | Carbon NPs | Mukherjee et al | Small | 2015 |
| Complement | Inorganic | Silicon | Muthusubramaniam et al | Annals of Biomedical Engineering | 2011 |
| Complement | Polymer-based | Polymers | Nie et al | Biomacromolecules | 2015 |
| Complement | Polymer-based | Polymeric NPs | Oommen et al | ACS Applied Materials and Interfaces | 2016 |
| Complement | Polymer-based | Polymeric NPs | Paillard et al | Pharmaceutical Research | 2010 |
| Complement | Inorganic | Graphene | Paul et al | Trends in Biomaterials and Artificial Organs | 2011 |
| Complement | Polymer-based | Polymers | Pereira et al | Toxicology in Vitro | 2015 |
| Complement | Lipid-based | Lipid NPs | Pham et al | Journal of Biological Chemistry | 2011 |
| Complement | Inorganic | Carbon nanotubes | Pondman et al | Nanomedicine: Nanotechnology, Biology, and Medicine | 2015 |
| Complement | Inorganic | Carbon nanotubes | Pondman et al | Nanomedicine: Nanotechnology, Biology, and Medicine | 2014 |
| Complement | Polymer-based | Polymeric NPs | Quaglia et al | International Journal of Pharmaceutics | 2006 |
| Complement | Polymer-based | Micelles | Quaglia et al | Journal of Biomedical Materials Research-Part A | 2008 |
| Complement | Lipid-based | Lipid NPs | Resnier et al | International Journal of Pharmaceutics | 2013 |
| Complement | Lipid-based | Lipid NPs | Resnier et al | Biotechnology Journal | 2014 |
| Complement | Inorganic | Carbon nanotubes | Rybak-Smith et al | Journal of Biomedical Nanotechnology | 2011 |
| Complement | Polymer-based | Hydrogels | Saini et al | Acta Biomaterialia | 2016 |
| Complement | Inorganic | Carbon nanotubes | Salvador-Morales et al | Molecular Immunology | 2006 |
| Complement | Lipid-based | Lipid NPs | Salvador-Morales et al | Biomaterials | 2009 |
| Complement | Polymer-based | Polymeric NPs | Salvador-Morales et al | Biomaterials | 2009 |
| Complement | Inorganic | Gold NPs | Sasidharan et al | ACS Biomaterials Science and Engineering | 2016 |
| Complement | Polymer-based | Polymeric NPs | Shan et al | Biomedical Microdevices | 2009 |
| Complement | Polymer-based | Polymeric NPs | Shenoi et al | Journal of the American Chemical Society | 2012 |
| Complement | Polymer-based | Polymeric NPs | Socha et al | Journal of Drug Targeting | 2009 |
| Complement | Polymer-based | Polymeric NPs | Sun et al | Analytica Chimica Acta | 2013 |
| Complement | Lipid-based | Liposomes | Szebeni et al | Nanomedicine: Nanotechnology, Biology, and Medicine | 2012 |
| Complement | Lipid-based | Liposomes | Szebeni et al | Journal of Controlled Release | 2012 |
| Complement | Inorganic | Graphene oxide | Tan et al | ACS Applied Materials and Interfaces | 2013 |
| Complement | Inorganic | Silica NPs | Tenzer et al | Nature Nanotechnology | 2013 |
| Complement | Polymer-based | Polymeric NPs | Tong et al | Journal of Materials Chemistry B | 2013 |
| Complement | Polymer-based | Polymeric NPs | Trung Bui et al | Biomacromolecules | 2013 |
| Complement | Polymer-based | Polymeric NPs | Vauthier et al | Biomaterials | 2011 |
| Complement | Polymer-based | Polymeric NPs | Vedakumari et al | Biochimica et Biophysica Acta - General Subjects | 2013 |
| Complement | Inorganic | Iron oxide NPs | Verhoef et al | Biomaterials | 2017 |
| Complement | Lipid-based | Lipid NPs | Vonarbourg et al | Journal of Biomedical Materials Research-Part A | 2006 |
| Complement | Polymer-based | Polymers | Wang et al | Journal of Materials Chemistry B | 2015 |
| Complement | Lipid-based | Liposomes | Wibroe et al | Journal of Controlled Release | 2016 |
| Complement | Polymer-based | Polymeric NPs | Xu et al | Small | 2016 |
| Complement | Polymer-based | Polymers | Xu et al | RSC Advances | 2015 |
| Complement | Polymer-based | Polymeric NPs | Yang et al | Journal of Nanoscience and Nanotechnology | 2010 |
| Complement | Lipid-based | Lipid bilayer | Yorulmaz et al | Colloids and Surfaces B: Biointerfaces | 2016 |
| Complement | Inorganic | Gold NPs | You et al | Particle and Fibre Toxicology | 2014 |
| Complement | Polymer-based | Polymeric NPs | Yousefi et al | Pharmaceutical Research | 2014 |
| Complement | Lipid-based | Lipid NPs | Yousefi et al | Pharmaceutical Research | 2014 |
| Complement | Polymer-based | Polymeric NPs | Yu et al | ACS Nano | 2014 |
| Complement | Polymer-based | Polymeric NPs | Zarrabi et al | Journal of Materials Science: Materials in Medicine | 2014 |
